# Epigenome-wide DNA methylation regulates cardinal pathological features of psoriasis

**DOI:** 10.1186/s13148-018-0541-9

**Published:** 2018-08-09

**Authors:** Aditi Chandra, Swapan Senapati, Sudipta Roy, Gobinda Chatterjee, Raghunath Chatterjee

**Affiliations:** 1 Human Genetics Unit, Indian Statistical Institute, 203 B. T. Road, Kolkata, West Bengal 700108 India; 2 Uttarpara, Hooghly, West Bengal 712258 India; 3 MDDC, Lansdowne Place, Kolkata, West Bengal India; 4 Department of Dermatology, IPGMER/SSKM Hospital, Kolkata, West Bengal India

**Keywords:** Differentially methylated probes, Methylation-sensitive PCR, Bisulfite cloning and sequencing, Gene expression, Histopathology of psoriasis, Munro’s microabscess, Rete peg elongation, Kogoj’s abscess

## Abstract

**Background:**

Psoriasis is a chronic inflammatory autoimmune skin disorder. Several studies suggested psoriasis to be a complex multifactorial disease, but the exact triggering factor is yet to be determined. Evidences suggest that in addition to genetic factors, epigenetic reprogramming is also involved in psoriasis development. Major histopathological features, like increased proliferation and abnormal differentiation of keratinocytes, and immune cell infiltrations are characteristic marks of psoriatic skin lesions. Following therapy, histopathological features as well as aberrant DNA methylation reversed to normal levels. To understand the role of DNA methylation in regulating these crucial histopathologic features, we investigated the genome-wide DNA methylation profile of psoriasis patients with different histopathological features.

**Results:**

Genome-wide DNA methylation profiling of psoriatic and adjacent normal skin tissues identified several novel differentially methylated regions associated with psoriasis. Differentially methylated CpGs were significantly enriched in several psoriasis susceptibility (PSORS) regions and epigenetically regulated the expression of key pathogenic genes, even with low-CpG promoters. Top differentially methylated genes overlapped with PSORS regions including S100A9, SELENBP1, CARD14, KAZN and PTPN22 showed inverse correlation between methylation and gene expression. We identified differentially methylated genes associated with characteristic histopathological features in psoriasis. Psoriatic skin with Munro’s microabscess, a distinctive feature in psoriasis including parakeratosis and neutrophil accumulation at the stratum corneum, was enriched with differentially methylated genes involved in neutrophil chemotaxis. Rete peg elongation and focal hypergranulosis were also associated with epigenetically regulated genes, supporting the reversible nature of these characteristic features during remission and relapse of the lesions.

**Conclusion:**

Our study, for the first time, indicated the possible involvement of DNA methylation in regulating the cardinal pathophysiological features in psoriasis. Common genes involved in regulation of these pathologies may be used to develop drugs for better clinical management of psoriasis.

**Electronic supplementary material:**

The online version of this article (10.1186/s13148-018-0541-9) contains supplementary material, which is available to authorized users.

## Background

Psoriasis is a chronic inflammatory skin disease suggested to be mediated by complex interaction of genetic, epigenetic and environmental factors [1]. The disease prevalence is estimated to be between 0.2 and 11.8% across different population worldwide [2, 3]. It is characterised by hyper proliferation and abnormal differentiation of keratinocytes, manifested as distinct elevated dry red scaly plaques on skin surface. The epidermal changes are thought to be preceded by a faulty immune activation, mainly mediated by T cells [4]. Genome-wide association and linkage-based studies have identified several psoriasis susceptibility regions (PSORS) predisposing to the disease [1, 5–9]. However, many of them failed to be replicated in other populations, except HLA-Cw6 allele in patients with age of disease onset below 40 years [10–18].

Despite the different genetic backgrounds, basic phenotypic presentation of the disease remains essentially similar across different populations. Studies on monozygotic twins showed that disease concordance is about 35–72% [19], thereby indicating the involvement of epigenetics in addition to the genetic susceptibility factors. Among all epigenetic mechanisms, DNA methylation has been reported to be one of the important factors for keratinocyte differentiation [20, 21]. It has been reported that the commonly used drug in psoriasis, e.g. methotrexate, can interfere with methyl transfer function of folate, thereby reverting to normal methylation state [22]. The reversible and relapsing nature of the disease again indicates involvement of epigenetic anomalies in psoriasis pathogenesis [1].

Histopathologic features including abnormal retention of nuclei in keratinocytes of stratum corneum (parakeratosis), aggregation of neutrophils in the parakeratotic stratum corneum (Munro’s microabscess), unevenly thickened epidermis with elongated rete ridges, absence of granular layer (hypogranulosis) or locally thickened granular layer (focal hypergranulosis), occasional aggregation of neutrophils along with spongiosis in stratum spinosum (called spongiform pustules of Kogoj or Kogoj’s abscess), dilated and tortuous blood vessels in the dermis and perivascular collection of immune cells are frequently observed in psoriasis.

Recent studies on skin tissues and peripheral blood mononuclear cells (PBMCs) of psoriasis patients established significant role of DNA methylation in disease pathogenesis [23–33]. Aberrant methylation and inversely correlated expression was shown for genes including demethylation of promoter2 of SHP-1 or protein tyrosine phosphatase non-receptor 6 (PTPN6) [23], hypermethylation of p16 [24] and secreted frizzled-related protein 4 (SFRP4) [25], hypomethylation of inhibitor of DNA binding 4 (ID4) [26] and 2′-5′-oligoadenylate synthetase 2 (OAS2) [27]. Overexpression of DNA methyltransferase 1 (DNMT1) and global hypermethylation was also reported in psoriatic tissues and PBMCs of psoriasis patients [28]. LINE-1 repeat elements were reported to be hypomethylated disrupting neighbouring gene expression in psoriatic tissue [29]. Genome-wide DNA methylation studies showed aberrant methylation in psoriatic skin tissue [30–33], which was reversed back to normal upon treatment [30, 33].

Several studies have reported reversal of aberrant DNA methylation [30, 33] as well as resolving of histopathological features associated with psoriasis following therapy [34]. However, there had not been any report addressing if there is any association between the altered DNA methylation and crucial histopathological features observed in psoriasis. We have shown for the first time that DNA methylation regulates the expression of genes associated with key histopathologic features of psoriasis. We also report genome-wide DNA methylation alterations between psoriatic and adjacent normal skin tissue for the first time in Indian population. We report here that aberrant DNA methylation in psoriatic skins was significantly enriched in several PSORS regions and may affect the expression of key pathogenic genes. We further show that DNA methylation at the CpG-poor promoters can inversely regulate downstream gene expression.

## Results

### Characterisation of the differentially methylated probes (DMPs)

**Fig. 1:**
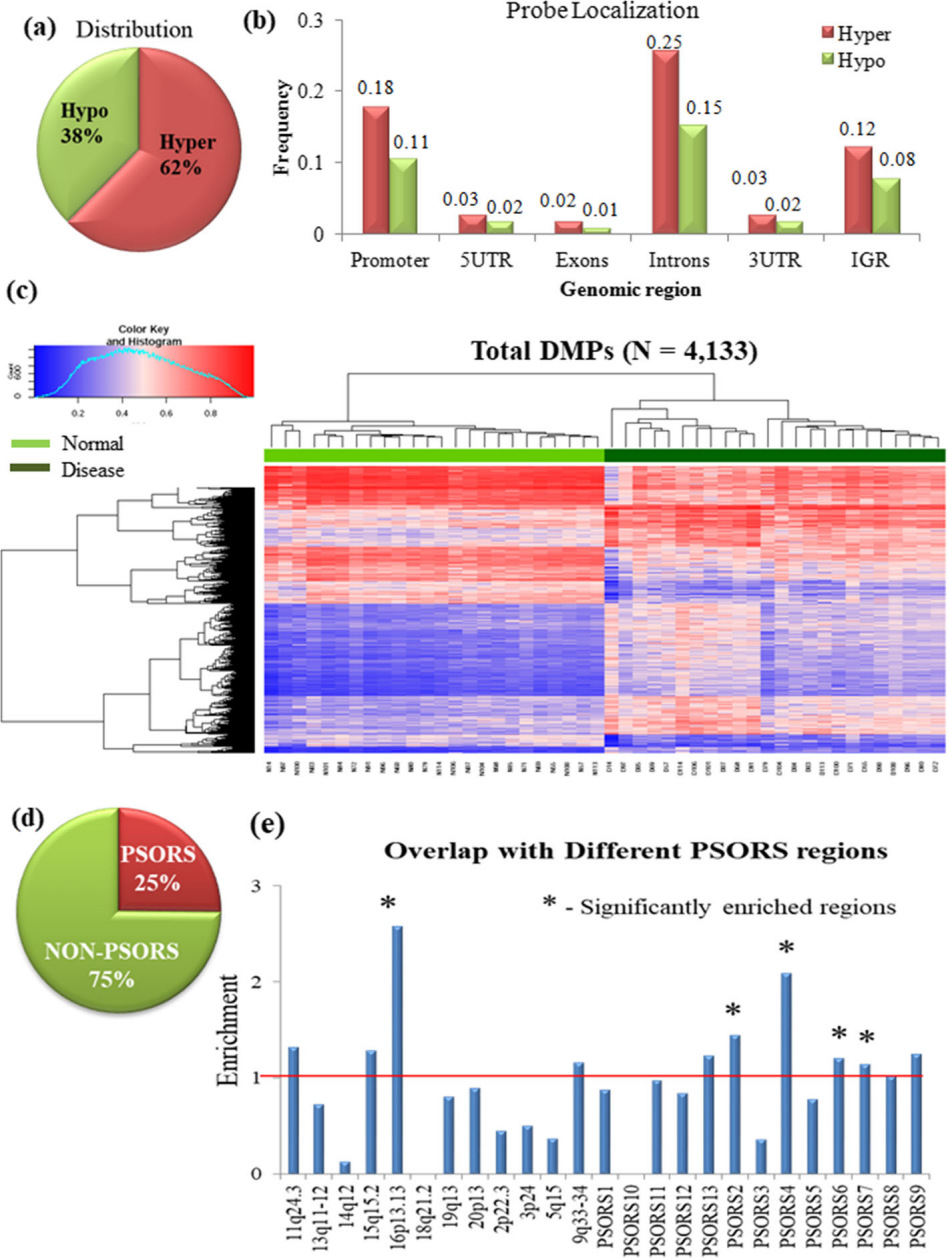
Characterisation of differentially methylated probes (DMPs) (*N* = 4133). **a** Classification of the DMPs into hyper- and hypomethylated probes. **b** Distribution of the hyper- and hypomethylated probes across different genomic regions. **c** Hierarchical clustering with 4133 DMPs shows distinctly separate clusters for disease and adjacent normal samples. Normal samples are marked in light green, and disease samples are marked in dark green. **d** Overlap of DMPs with psoriasis susceptibility regions (PSORS) and non-PSORS. **e** Distribution of PSORS-overlapping DMPs across various known PSORS loci. Loci significantly enriched (*P* value ≤ 0.05) in hypergeometric test are marked (*)

We conducted a genome-wide DNA methylation profiling of psoriatic and adjacent normal skin tissues from 24 patients belonging to Eastern Indian population (Table 1). Differentially methylated probes (DMPs) were identified using *β* values that were at least 15% differential (|Δ*β*| > 0.15) between disease and adjacent normal tissues with false discovery rate (FDR)-adjusted *P* value ≤ 0.05. In total, 4133 CpG sites were differentially methylated in disease compared to adjacent normal tissues (Additional file 1). Approximately 62% (*N* = 2578) of the DMPs were hypermethylated, while only 38% (*N* = 1555) were hypomethylated in disease tissues. Genomic distribution of the DMPs showed that they were mainly enriched in the introns, followed by promoters and intergenic regions (Fig. 1a,b). The distribution was similar for both hyper- and hypomethylated DMPs (Additional file 2: Figure S1a). Hypermethylation in all these regions is more prevalent than hypomethylation (Fig. 1b), as also observed previously [31], suggesting a trend towards global hypermethylation in psoriatic tissues. Unsupervised hierarchical clustering showed distinct classification of normal and disease samples (Fig. 1c). Highest enrichment of DMPs was observed in intronic regions (41%), followed by promoters (28%). Note that the 450k array design includes highest number of probes in the promoters, followed by intron and intergenic regions (Additional file 2: Figure S1b). Around 11% of the DMPs were overlapped with repeat elements, and most of these DMPs were primarily enriched in the introns (45%) (Additional file 2: Figure S1c-d). Contrary to the overall distributions, DMPs overlapped with the repeat elements had higher frequency of hypomethylated CpGs than the hypermethylated CpGs (Additional file 2: Figure S1c). LINE elements contain largest proportion of hypomethylated sites (Additional file 2: Figure S1e).

**Table 1:** Demographic characteristics of discovery and validation cohort samples

Study subjects	Discovery cohort: 48 (24 paired) samples	Validation cohort: 30 (15 paired) samples
Mean age (in years)	37.94 (SD = 14.24) (range 12–74)	39.83 (SD = 10.77) (range 22–60)
Mean age of onset (in years)	33.17 (SD = 14.03) (range 10–73)	33.12 (SD = 11.21) (range 9–52)
Type I–type II	19–5	10–5
Type I–type II: age distribution	Mean age/onset 32.4/28.3	Mean age/onset 34.05/26.83
	Mean age/onset 54/51.5	Mean age/onset 51.4/45.7
Sex distribution	18 males, 6 females	10 males, 5 females

Even though the majority of differential sites (58%) were in CpG-poor open sea regions, CpG islands (CGIs) and associated regions (CGI shores and shelves) showed higher degree of hypermethylation (47%) than hypomethylation (35%) (Additional file 2: Figure S2a-b). Unlike hypomethylated CGI shores, hypermethylated CGI shores were significantly differentially methylated, while shelves were not differential at all (Additional file 2: Figure S2c).

In order to identify the involvement of DNA methylation in regulating genes within psoriasis susceptibility regions, we determined DMPs that were overlapped with PSORS regions. A significant proportion (*P* value 5.1 × 10^−3^) of DMPs (*N* = 1040) were overlapped with the PSORS loci (Fig. 1d). Significant enrichment was observed for PSORS2, PSORS4, PSORS6, PSORS7 and locus at 16p13.13 (Fig. 1e). On closer look at the differential sites, we observed that several of them were located at promoters of known psoriasis susceptibility genes. This include promoters of the shorter isoform of caspase recruitment domain family member 14 (CARD14) (in PSORS2) and several S100 calcium-binding genes (S100A3, S100A5, S100A13) in epidermal differentiation complex (in PSORS4), which were hypermethylated, while others, e.g. S100A8, S100A9 and S100A12, were hypomethylated. The promoter region of protein tyrosine phosphatase non-receptor type 22 (PTPN22) (in PSORS7) showed significant hypomethylation. The detailed list of PSORS associated genes that showed differential methylation is presented in Additional file 3.

### Differential methylation in promoter regions

We next identified the differentially methylated promoters and evaluated their downstream gene regulation. Approximately 29% (*N* = 1168) of the DMPs were overlapped with 764 promoters (Additional file 4). Hierarchical clustering using differentially methylated promoters classified normal and disease samples into two distinct clusters (Additional file 2: Figure S3a). Similar to the DMPs, 59% (*N* = 451) of the differentially methylated promoters were hypermethylated, while the remaining 41% (*N* = 313) were hypomethylated. Around 57% of hypermethylated promoters overlapped with CGI or associated regions, whereas only 33% hypomethylated promoters were overlapped with CGIs (Additional file 2: Figure S3b). Gene ontology (GO) analysis with the differentially methylated promoters showed enrichment of biological processes including regulation of immune system process, T cell activation, regulation of cell adhesion, regulation of leukocyte activation, inflammatory response, leukocyte and neutrophil migration among the top enriched processes (Table 2, Additional file 5: Table S1). Several genes reported to be involved in psoriasis pathogenesis including S100A9, S100A8, PTPN22, PTPN6, LAMA4, IL1B and IL12RA were involved in many of these enriched biological processes (Additional file 5: Table S1). On classification into hyper- and hypomethylated promoters, the hypermethylated promoters showed enrichment of biological processes including cellular development and differentiation, actin cytoskeletal organisation, cell adhesion and motility, while hypomethylated promoters showed enrichment of immune-activation processes including inflammation, T cell activation, cytokine production and cellular proliferation (Additional file 6: Table S1).

**Table 2:** List of top enriched biological processes identified from gene ontology study with the differential promoters

GO term: biological process	Gene count (%)	Fold enrichment	*P* value (BH)
Regulation of immune system process	14.11	2.11	4.40 × 10^−9^
T cell activation	6.31	2.91	4.90 × 10^−7^
Regulation of cell adhesion	7.81	2.50	6.89 × 10^−7^
Regulation of leukocyte activation	5.71	2.51	4.38 × 10^−5^
Inflammatory response	7.66	2.48	1.11 × 10^−6^
Cell migration	12.76	2.22	5.20 × 10^−9^
Regulation of cell migration	7.21	2.17	7.15 × 10^−5^
Leukocyte migration	5.40	2.96	2.77 × 10^−6^
Neutrophil migration	2.40	5.26	2.86 × 10^−5^
Positive regulation of MAPK cascade	5.56	2.36	1.85 × 10^−4^
Extracellular matrix organisation	3.90	2.41	3.43 × 10^−3^

### Validation and downstream effect of promoter methylation

**Fig. 2:**
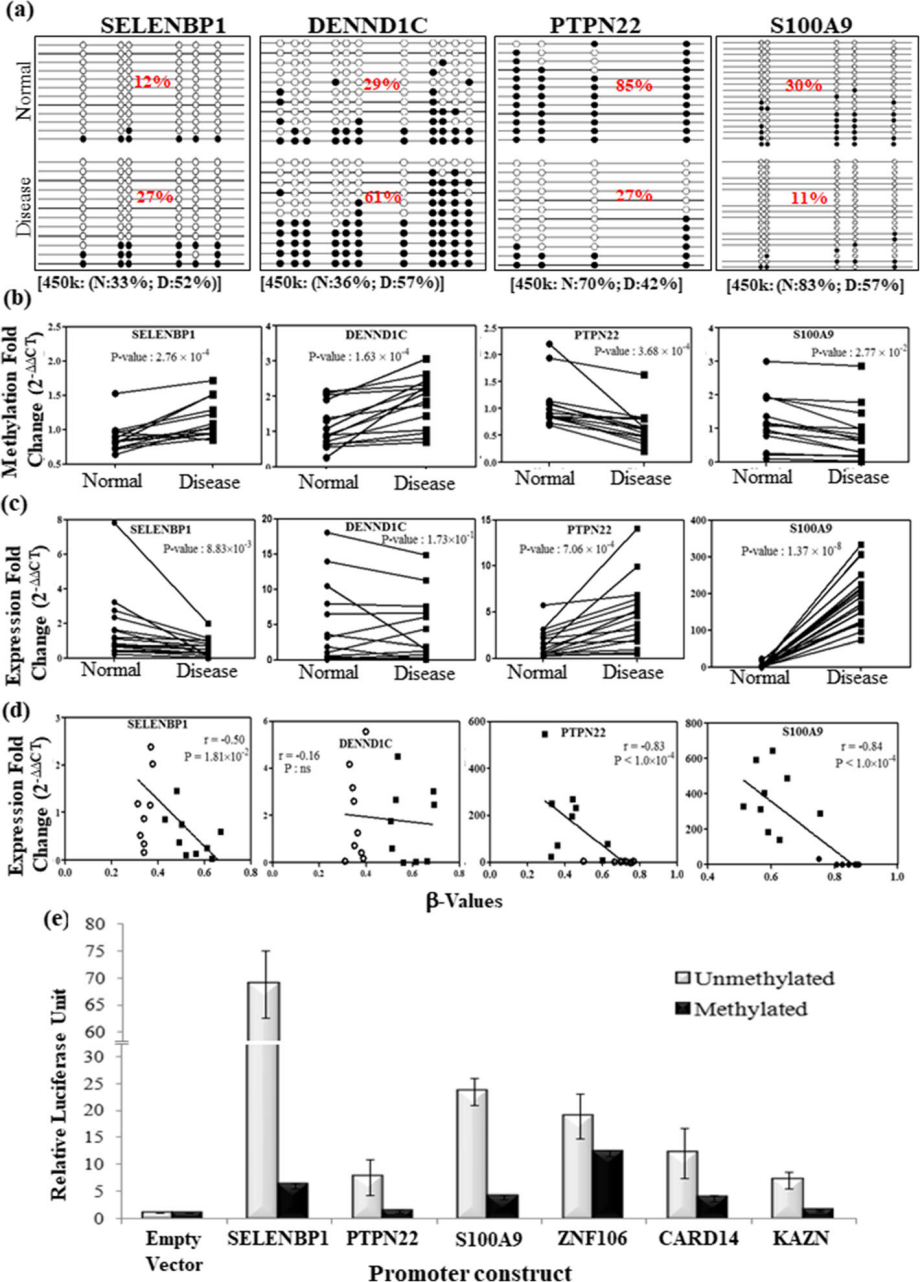
Validation of differentially methylated promoters in an additional independent set of samples**. a** Graphical representation of cloning and sequencing of BSP products from adjacent normal and disease samples, for four selected promoter regions. The average promoter methylation (%) for the normal and disease samples are presented inside the figure. Average *β* values (%) for these selected promoters are presented at the bottom of the figure. **b** Validation of promoter methylation status in paired disease and adjacent normal samples through quantitative methylation-sensitive PCR (qMSP). **c** Gene expression fold change in disease and adjacent normal samples of corresponding genes. **d** Scatter plot showing inverse correlation between methylation level (*β* value) and gene expression fold change. **e** Relative luciferase expression of the unmethylated and methylated versions of the promoter constructs for selected genes. *P value ≤ 0.05

Differentially methylated promoters, obtained from the genome-wide array data, were validated in an independent set of paired disease and adjacent normal samples (Table 1). For the validation study, we included 10 promoters that had at least 3 DMPs. Among these, 8 promoters were overlapped with PSORS regions, while zinc finger protein (ZNF106) and signalling lymphocytic activation molecule family member 1 (SLAMF1) were outside PSORS regions. Cloning and sequencing of the bisulfite-converted products showed concordant results with genome-wide array data. Similar degree of hypermethylation for selenium-binding protein (SELENBP1), DENN domain containing 1C (DENND1C) and hypomethylation for S100A9, PTPN22 were observed (Fig. 2a). Differential methylation of these 10 promoters was also validated using bisulfite sequencing PCR (BSP) followed by qMSP. Around 80–100% of the samples showed similar differential methylation as observed in genome-wide data (Fig. 2b, Additional file 2: Figure S3c).

To determine the effect of differential promoter methylation, we checked the downstream gene expression of these selected promoters in paired disease and adjacent normal tissue samples. Significant downregulation for hypermethylated genes SELENBP1 and ZNF106 and significant upregulation for hypomethylated genes PTPN22 and S100A9 were observed in psoriatic tissues compared to adjacent normal (Fig. 2c, Additional file 2: Figure S3d). CARD14 and DENND1C also showed overall downregulation, but did not reach the level of significance at 0.05 (Fig. 2c, Additional file 2: Figure S3d). Note that there are other larger isoforms of these two genes which do not show differential promoter methylation. As the smaller isoforms had no unique exons, we could not exclusively check expression of those isoforms which harboured differentially methylated promoter. Average promoter methylation (*β* value) and corresponding gene expression (fold change) showed significant inverse correlation for SELENBP1 (*r* = − 0.5), PTPN22 (*r* = − 0.83) and S100A9 (*r* = − 0.84), except for DENND1C (*r* = − 0.16) (Fig. 2d).

Furthermore, in order to substantiate that the expression of these genes were actually controlled by the methylation states of their promoters, we cloned six CpG-poor promoters (SELENBP1, PTPN22, S100A9, ZNF106, CARD14, KAZN) into a CpG-less luciferase reporter vector (pCpGL) (Fig. 2e). The CpGs within the cloned promoter regions of pCpGL-promoter constructs were enzymatically methylated (Additional file 2: Figure S4), and both methylated and unmethylated plasmids were transiently transfected to the HEK293T cells. Unmethylated promoters were significantly overexpressed, while the methylated version abrogated the luciferase expression (Fig. 2e), suggesting that their aberrant expression in disease might be mediated by differential DNA methylation in psoriasis.

### Epigenetic regulation of histopathological features of psoriasis

Next, we classified the patients based on the presence of key histopathologic features including Munro’s microabscess, Kogoj’s microabscess, elongation of rete pegs and focal hypogranulosis and determined the unique differentially methylated CpGs in the psoriatic skins associated with each of these features (Additional file 2: Figure S5, Table 3).

**Table 3:** Histopathological characterisation of the discovery cohort samples

Histopathological parameter	Characteristics
Parakeratosis	Present: confluent- 19, focal- 3; NA: 3
Focal hypergranulosis	Present- 7, absent- 17
Munro’s microabscess	Present- 11, absent- 10, NA- 3
Kogoj’s microabscess	Present- 9, absent- 15
Elongation of rete pegs	Mild- 6, moderate- 14, severe- 4

**Fig. 3:**
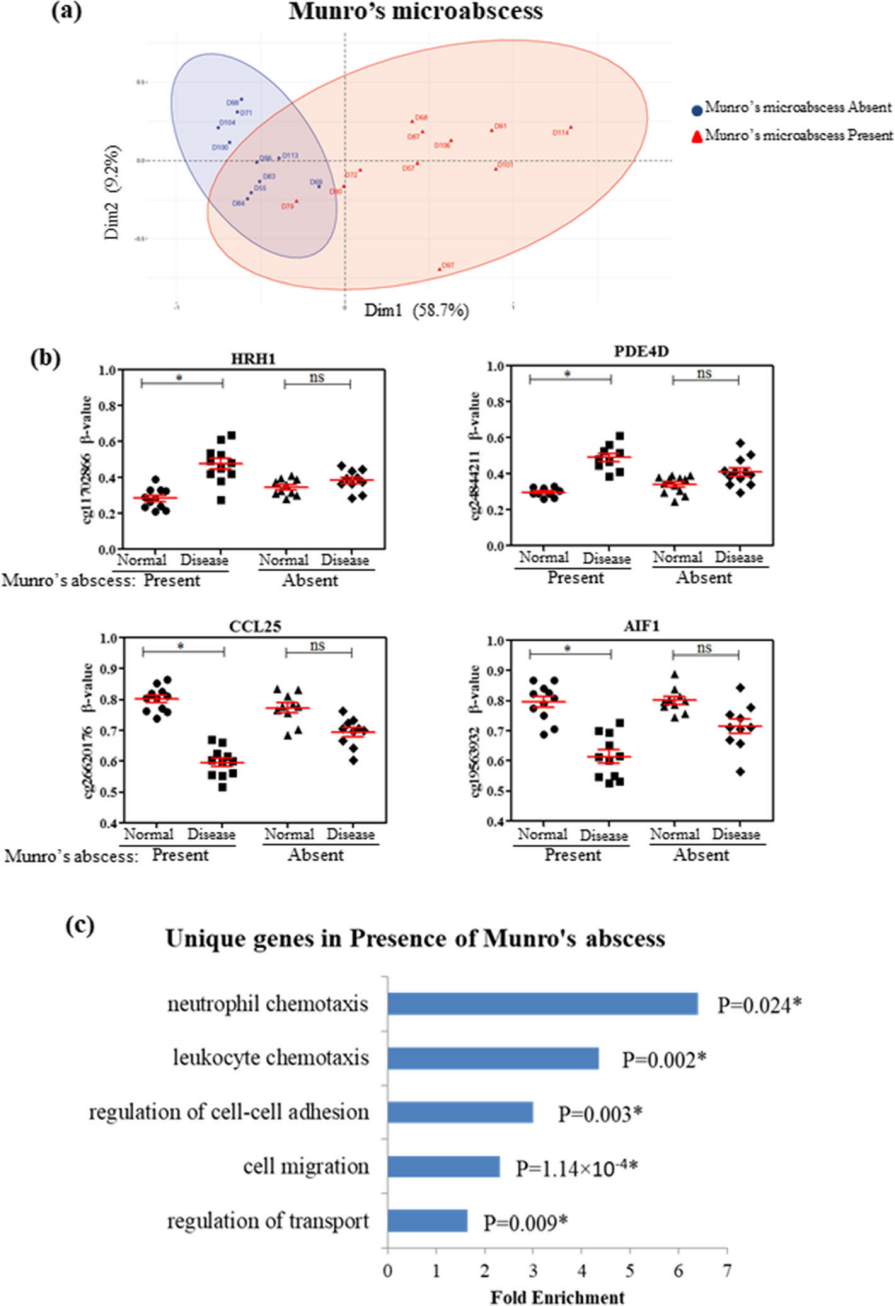
DNA methylation in regulation of Munro’s microabscess formation. **a** Principal component analysis (PCA) with top 100 differential CpG sites unique for Munro’s abscess present samples shows distinct clusterings for samples with and without Munro’s microabscess. **b** Candidate promoter-overlapping sites that are differential only in samples with Munro’s microabscess but not in the other group. **c** Gene ontology study with gene promoters overlapping with unique DMPs. *P value ≤ 0.05

Significant DMPs were identified between psoriatic skin and adjacent normal tissues for the groups with and without Munro’s microabscess (Table 3). We identified 831 and 48 DMPs that were uniquely differential in Munro’s microabscess present and absent groups, respectively (Additional file 7). Around 1508 DMPs were commonly differential in both groups (Additional file 7). Principal component analysis (PCA) with top unique DMPs showed distinct clustering of the two groups (Fig. 3a, b). To determine the genes that might be regulated by these unique DMPs, GO analysis was performed with the promoters that were overlapped with these unique DMPs. Interestingly, the highest enrichment was observed for the neutrophil and leukocyte chemotaxis processes (Fig. 3c), indicating the epigenetic regulation of genes involved in Munro’s microabscess. These processes include genes like histamine receptor H1 (HRH1), phosphodiesterase 4D (PDE4D), C-C motif chemokine ligand 25 (CCL25) and allograft inflammatory factor 1 (AIF1) (Fig. 3b). A disintegrin and metalloproteinase domain-containing protein 10 (ADAM10), free fatty acid receptor 2 (FFAR2), interleukin 1 beta (IL1B) and triggering receptor expressed on myeloid cells 1 (TREM1), involved in neutrophil and leukocyte chemotaxis, were also differentially methylated in the psoriatic skin tissues of patients with Munro’s microabscess (Additional file 5: Table S2).

**Fig. 4:**
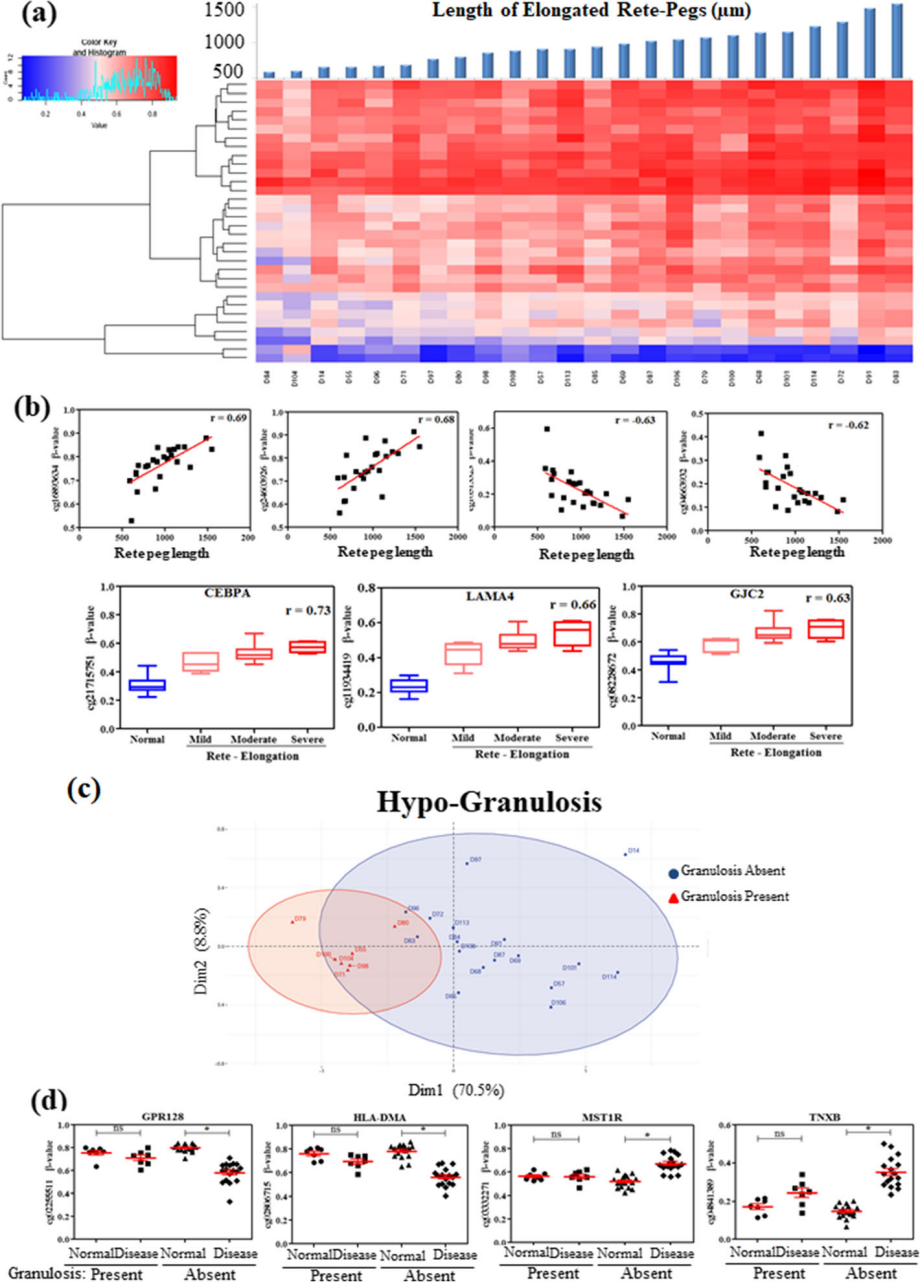
Involvement of DNA methylation in regulation of rete peg elongation and focal hypergranulosis. **a** Heatmap of 60 highly correlated sites (*R* > 0.7) with rete peg length. **b** Candidate sites showing inverse or direct correlation between *β* values and rete peg length; gene promoters showing correlation with normal as well as grades of rete peg length. **c** PCA with top 100 unique DMPs classifying samples based on occurrence of focal hypergranulosis. **d** Candidate promoter-overlapping sites that are differential only in samples without focal hypergranulosis but not in the other group. *P value ≤ 0.05

Maximum length of the elongated rete pegs was measured from each patient. Correlation between maximum rete peg lengths and methylation status (*β* value) of the differential CpG sites were studied. We observed 391 CpG sites that showed significant correlation of methylation level with the rete peg length in the psoriatic skin (*P* value ≤ 0.05 and │*r* ≥ 0.4) (Additional file 8); of these, 32 DMPs showed relatively high correlation (│*r*│ ≥ 0.6) (Fig. 4a). Nearest genes to these DMPs include CCAAT/enhancer binding protein alpha (CEBPA), laminin subunit alpha 4 (LAMA4) and gap junction protein gamma 2 (GJC2) (Fig. 4b; Additional file 2: Figure S6a, b; and Additional file 8).

Due to rapid proliferation and incomplete differentiation of psoriatic keratinocytes, the granular layer is absent in disease tissue. The granules appear prematurely in some samples leading to focal hypergranulosis. Analysing the samples with and without focal hypergranulosis identified 4450 unique DMPs in samples without focal hypergranulosis, while only 20 DMPs were unique in samples with focal hypergranulosis (Additional file 9). First two principal components of the PCA explained 79.3% of variations between these two groups (Fig. 4c). Genes with unique differentially hypomethylated promoters include G protein-coupled receptor 128 (GPR128) and human leukocyte antigen (HLA)-DMA, while hypermethylated promoters include macrophage stimulating 1 receptor (MST1R) and tenascin-XB (TNXB) in patients without focal hypergranulosis (Fig. 4d, Additional file 9).

**Fig. 5:**
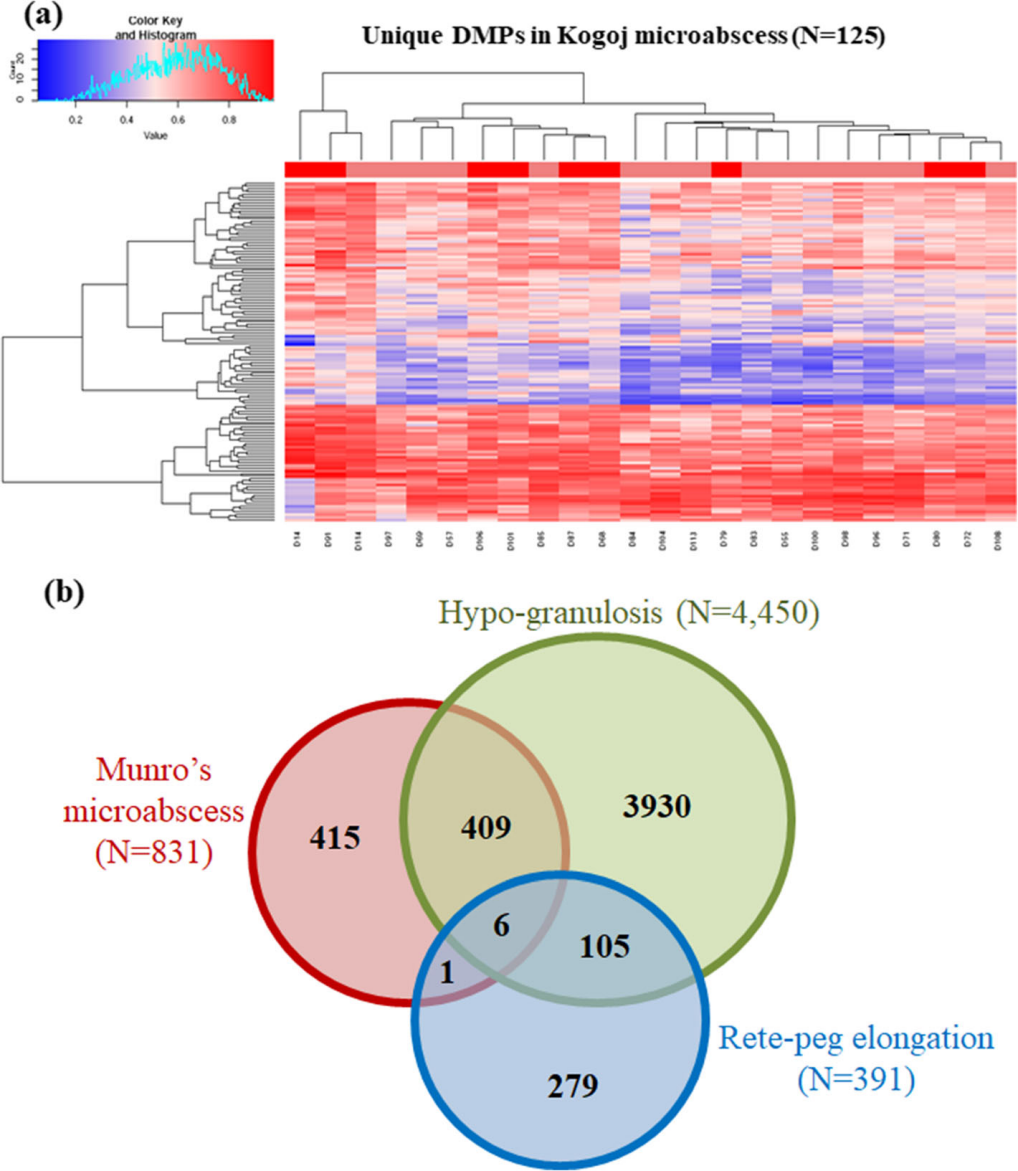
**a** Heatmap showing clustering of samples with and without Kogoj’s microabscess. Unique DMPs identified only in the presence of Kogoj’s microabscess could not classify between samples with the abscess (marked in dark red) and those without it (marked in light red) into separate clusters. **b** Venn diagram representing overlap of unique DMPs identified separately for each histopathological feature: Munro’s microabscess, hypogranulosis and rete peg elongation

Another characteristic feature, Kogoj’s microabscess was detected in 9 samples, while remaining 15 samples did not show any sign of this histopathologic feature (Table 3, Additional file 2: Figure S5). On comparison of samples classified based on the presence of Kogoj’s abscess, we identified 125 unique DMPs in samples containing this abscess, while only 25 DMPs were unique in the group without Kogoj’s abscess (Additional file 10). These unique DMPs for the present and absent groups were mapped into 25 and 8 promoters, respectively. GO analysis identified regulation of transport and stress response processes for these genes. Nevertheless, the identified unique sites did not show separate clustering of the samples with and without Kogoj’s microabscess (Fig. 5a).

Finally, we sought to identify if there are any common DMPs among the unique sets observed to be associated with different histopathological features. We identified 7 DMPs that were common between Munro’s microabscess and rete peg elongation, 111 common between hypogranulosis and rete peg elongation, and 415 common between hypogranulosis and Munro’s microabscess. However, only 6 hypermethylated probes were found to be common for all three characteristic histological features (Fig. 5b). Of these, 3 sites overlapped with promoters of synaptogyrin 1 (SYNGR1) (smaller isoform), NADH dehydrogenase 1 beta subcomplex 4 (NDUFB4) and testis-specific serine kinase 1B (TSSK1B), and two of the sites overlapped with PSORS loci.

## Discussion

We have conducted a genome-wide DNA methylation study among Indian psoriasis patients using Illumina Infinium Human Methylation 450k BeadChip. Twenty-four paired tissue samples were used as the discovery set. Using a cut-off of adjusted *P* value ≤ 0.05 and |Δ*β*| > 0.15, we identified 4133 DMPs associated with psoriasis. Around 40–50% DMPs, identified in previous studies [30, 32], which compared psoriatic and adjacent normal skin tissues, were also detected in our study. Precisely, 756 sites (including 440 hyper- and 316 hypomethylated DMPs) out of 1514 total differential sites, identified previously, were overlapped with DMPs identified in our study [32]. It is worth mentioning here that this study used 10% delta-beta cut-off (|Δ*β*| ≥ 0.1) and used Bonferroni correction to control for the multiple hypothesis tests [32]. Another study with Illumina Infinium Human Methylation 27k array, which used differential *M* values also showed ~ 44% (12 out 27 sites) overlap with our study [30]. Hierarchical clustering with the DMPs was able to accurately classify the normal and disease samples (Fig. 1c), again supporting the role of epigenetic alterations during psoriasis pathogenesis.

Differential sites were mostly enriched in intronic regions, as also observed previously [31]. Introns are known to harbour different regulatory regions like enhancer elements, which might lead to an additional level of gene regulation associated with psoriasis; however, these remain mostly uncharacterised in context of psoriasis. Hypomethylation of LINE elements, observed in our study, is also supported by previous reports [29]. Higher proportion of hypermethylated probes was also commonly observed in previous studies [30, 31], while opposite result was obtained when the epidermal part was separately studied [33]. Since the epidermis is mostly enriched with keratinocytes, and LINE-1 hypomethylation was found to be restricted only to psoriatic keratinocytes [29], this can explain the opposite global methylation pattern in the epidermis compartment. Global hypermethylation of psoriatic CD4+ T cells [35], dermal fibroblasts and contribution of other cell types, when total psoriatic skin tissues were considered, might have led to the global hypermethylation observed in our study as well as in previous studies [30, 31].

A subset of the genome-wide data was validated in an additional 15 pairs of samples using sodium bisulfite conversion followed by either sequencing of the cloned bisulfite converted product, or by quantitative methyl sensitive PCR (qMSP). Validation of some of these DMPs showed 80–100% concordance with the discovery set of samples. One may, in principle, argue to increase the |Δ*β*| cut-off to reduce the false positive rates, but simultaneously, it may also lead to missing out of the true positive signals. In the genome-wide data, when we considered |Δ*β*| cut-off > 0.3, we identified 60 DMPs, of which only 11 sites overlapped with promoters. With a |Δ*β*| cut-off > 0.2, we identified 861 DMPs (Additional file 1). In our validation study using cloning and sequencing of the bisulfite converted products, we considered 7 DMPs with |Δ*β*| between 0.15 and 0.2, 5 DMPs with |Δ*β*| between 0.2 and 0.3 and 3 DMPs with |Δ*β*| > 0.3. We observed a concordance of 80–100% for all these probes. qMSP validation in 15 additional samples comprising 38 DMPs, of which 35 had |Δ*β*| < 0.3, also showed similar results, thus confirming the reliability of DMPs obtained from the genome-wide data, and also added support to the |Δ*β*| cut-off of 0.15. We have considered total skin biopsy samples of psoriasis patients, which is a heterogeneous collection of different cell types. Number of cells of a particular cell type may also vary between normal and diseased tissues. However, due to different methylation levels of different cell types, actual differential methylation might be averaged out and missed at higher cut-offs. We have shown that these DMPs could generate distinct clusters for the normal and disease samples, thereby demonstrating the crucial role of DNA methylation in psoriasis pathogenesis (Fig. 1c). A subset of these sites showed intermediate methylation, at least in half of the samples, which might be the reflection of intermediate state of the disease or a distinct clinical sub-type of the disease. Furthermore, a high correlation of CpG methylation with the rete peg elongation is clearly evident from our analysis. Rete peg elongation increases with the state of this disease, which also indicates the possible involvement of CpG methylation with the disease progression.

For the first time, we have identified involvement of DNA methylation and key pathogenic genes that might regulate the characteristic histopathological features observed in psoriasis. Gene expression study showed inverse correlation with promoter methylation status for most of the genes, which was also evident from previous studies [19, 30, 31]. Additionally, cloning the CpG-poor promoters into CpG-less (pCpGL) luciferase vector further established the fact that only altering methylation levels of the promoter can significantly regulate the downstream gene expression (Fig. 2e). Our study showed the direct proof of silencing of gene expression through promoter methylation in case of psoriasis. Although previous studies used pGL3 vectors to demonstrate this phenomenon, but methylation of CpG sites in this vector backbone might have interfered with the results obtained [35].

Some studies have focused on psoriatic PBMCs [19, 28, 35], epidermis [33] or dermal compartments only [36], we, however, wanted to include both the epidermal and dermal compartments along with infiltrating immune cells, as all of these cell types contribute to the disease. Since DNA methylation levels can be influenced by environmental factors, we had not included unrelated healthy controls in our analysis, whose different environmental exposure or unalike genetic background might add unnecessary differences thereby affecting data quality. In order to minimise influence of other factors, we thus compared methylation pattern of disease tissue with corresponding adjacent normal tissues from each patient. Both psoriatic and adjacent normal tissues were histopathologically analysed before considering for the genome-wide or validation studies.

Most interestingly, we observed overlapping of ~ 25% of the DMPs with PSORS loci (Fig. 1d), previously known psoriasis susceptibility regions conferring genetic predisposition to the disease. Prominent differential methylation was observed in promoter of several S100A genes within epidermal differentiation complex (EDC) located in PSORS4 (Additional file 6), complying with previous studies which also observed similar enrichment [30, 32]. Overexpression of S100A8-S100A9 has been reported in psoriatic tissue [37] and serum samples [38] as well, suggesting their prominent role in psoriasis pathogenesis. PTPN22, one of the top hypomethylated genes overlapping with PSORS7, showed inverse correlation of methylation with gene expression in our study. Although genetic association of this gene has been reported in psoriasis [39] as well as in other autoimmune diseases [40], there are no reports on its regulation by promoter methylation in psoriasis. Another top hypermethylated gene located in PSORS4, SELENBP1, has been identified in our study. This gene has not previously been reported in psoriasis. However, lower selenium levels observed in psoriasis patients [41] could be attributed to lowered expression of this gene. This suggests SELENBP1 as a potential candidate for studying the role of this gene and supplementation of this trace element in disease. Since both histopathological features and DNA methylation aberrations sometimes resolved on therapy, we wanted to study if DNA methylation could regulate these features. Due to the cell-type heterogeneity in different histopathological features, the results of differential methylation analysis are sometimes biased, which in turn increases the false positive results. Adjusting for the major cell-type compositions from the histopathological data may partially reduce the rate of false positives, however, that may substantially reduce the true positive predictions too. Here, to control the false positive rate, we have used a differential methylation threshold of |*∆β*| > 0.15 between disease and adjacent normal tissue and a FDR-adjusted *P* value ≤ 0.05. Comparing the differences in methylation with respect to different histopathological features, a unique set of DMPs were observed from paired analysis of samples with Munro’s microabscess, focal hypergranulosis and Kogoj microabscess. GO analysis with the unique differential promoters involved in Munro’s abscess formation revealed neutrophil and leukocyte chemotaxis as the highest enriched biological processes which include genes like HRH1, PDE4D, CCL25, AIF1, ADAM10, FFAR2, IL1B and TREM1. HRH1 has been reported to promote keratinocyte proliferation, suppression of differentiation and wound healing in keratinocytes [42, 43]. IL1B has also been previously reported in proliferation and intra-epidermal microabscess in flaky skin mice [44]. Abnormal inflammation, infiltration and accumulation of neutrophils in stratum corneum, as observed in Munro’s microabscess, thus, might be regulated by methylation, at least in part. This observation also adds support to our selected *∆β* cut-off value, as differential methylation was not enriched on genes that are expressed by neutrophils, but with those that are involved in neutrophil chemotaxis, a feature that is actually observed in disease. A set of highly correlated (|*r*| ≥ 0.6) methylated CpGs was identified with the rete peg elongation. Hypermethylated CpGs were observed in the promoter regions of C/EBPα, LAMA4, GJC2 and miR1178. C/EBPα has been reported to arrest cell proliferation through direct inhibition of CDK2 and CDK4 [45], while LAMA4 and GJC2 are involved in cell adhesion and differentiation. Increasing length of spinous layer with rete peg elongation thus can be attributed to the gradual hypermethylation and probable silencing of these genes.

Higher number of overlap of unique sites between Munro’s microabscess and focal hypogranulosis suggests their common causal feature, i.e. both are caused due to abnormal differentiation of keratinocytes. Interestingly, the common sites observed between all three histopathological features can be important targets for disease therapeutics. Since Kogoj’s abscess is a localised phenomenon, we could have easily missed particular sites containing this feature while selecting the site of biopsy. This might have limited the identification of actual genes that could regulate formation of Kogoj’s microabscess and could be the possible reason for misclassification of groups with and without Kogoj’s abscess. Studies from Indian population have also shown the inconsistent presence of this feature among psoriasis patients [46]. Regulation of histopathological features by DNA methylation is also established from the fact that after treatment, aberrant methylation levels revert back to normal and histopathological features resolve as well [30, 33, 34]. This observation further highlights the crucial role of DNA methylation in psoriasis pathogenesis.

## Conclusions

In conclusion, our study demonstrates the significant involvement of DNA methylation in psoriasis development. It appears to control disease progression as well as involved in manifestation of characteristic histopathological features. Since DNA methylation is reversible, this also explains the dynamic nature of the disease during remission and relapse of the psoriatic plaques. Identification of epigenetically regulated genes may be used to develop epigenetic drugs for disease management. Our study has identified a core set of DMPs and associated key molecules that are strongly involved in psoriasis pathogenesis, or presence of characteristic histopathologic features. Methylation status at these sites can be monitored and controlled to prevent disease occurrence or recurrence. These can thus be good targets for future clinical studies on psoriasis therapy. However, further validation and functional studies might be required to determine the precise clinically significant epigenetically regulated genes in psoriasis.

## Methods

### Study sample

Psoriasis patients (*N* = 39) were recruited from eastern region of India after obtaining written consent for participation in the study. Family history, comorbidities and other disease characteristics were recorded. Sample characteristics are summarised in Table 2. The disease was diagnosed by at least two dermatologists and confirmed by histopathological examination. Patients with generalised plaque type psoriasis were only included in the study to minimise clinical heterogeneity. Patients were kept without any systemic or topical therapy for at least 1 month prior to sample collection. Psoriatic and adjacent normal skin biopsies (4 mm) were obtained from each patient. A part of the sample was collected in formalin for histopathology; the remaining part was collected in RNA Later (Invitrogen) and stored at − 80 °C until further processing. The study was approved by the Institutional Ethics Committee for Human Research, Indian Statistical Institute, Kolkata, India and IPGMER, Kolkata, India, and conducted according to the Declaration of Helsinki Principles.

### DNA methylation study and data analysis

DNA was isolated from blood and skin tissues with DNeasy Blood and Tissue kit (Qiagen, Germany) using manufacturer’s instructions. Quality and concentration of each DNA sample was checked by Nanodrop Spectrophotometer (Nanodrop 2000). DNA isolated from 48 samples (24 psoriatic disease skin and 24 adjacent normal tissue) was subjected to Illumina Infinium Human Methylation 450k BeadChip, according to manufacturer’s protocol. Raw data (.idat files) obtained was analysed using Chip Analysis of Methylation Pipeline (ChAMP) available as R-Bioconductor package [47, 48]. After initial pre-processing, data were normalised using beta-mixture quantile (BMIQ) [49] method to correct for type 1 and type 2 probe bias. Batch effect removal was carried out using ComBat [50]. Illumina 450k bead array can profile 482,421 CpG sites, 3091 non-CpG sites and 65 random SNPs across the human genome [51, 52]. After removal of quality control probes, probes which failed to attain detection *P* value (cut-off ≤ 0.05), represented < 3 times in 5% samples, non-CpG probes, probes on sex chromosomes and those overlapping with polymorphic sites, we were left with 347,635 sites for downstream analysis. The batch-corrected *β* values were subjected to paired differential methylation analysis to identify differential probes. Differentially methylated probes (DMP) were identified using *β* values that were at least 15% differential (|Δ*β*| > 0.15) between disease and adjacent normal tissues with FDR-adjusted *P* value ≤ 0.05. The co-ordinates of the PSORS regions were identified by previous studies and reviewed earlier [1]. The overlap between DMPs and PSORS loci were identified using bedtools, and significance of locus-wise enrichment was carried out using hypergeometric test in R. Heatmaps were generated using Euclidean distance and complete linkage method using heatmap.2 function available in gplots package in R.

### Bisulfite sequencing PCR (BSP) and quantitative methylation-specific PCR (qMSP)

Promoters are defined in our study as 1500 bp regions, including 1000 bp upstream and 500 bp downstream from each transcription start site (TSS). We considered 10 promoters that had at least 3 DMPs for the validation. Among these, 8 promoters were overlapped with PSORS regions, while ZNF106 and SLAMF1 were outside PSORS regions. For validation of Illumina 450K methylation array data, 15 independent psoriatic and adjacent normal skin biopsy pairs were used (Table 1). Bisulfite treatment of the isolated DNA was carried out using EZ DNA Methylation Gold Kit (Zymo Research), according to manufacturer’s protocol. Converted DNA samples were used for bisulfite sequencing PCR (BSP) to amplify the selected promoter regions. BSP products were cloned into TA-cloning vector pTZ57R/T (Thermo Scientific) and transformed into *E. coli* JM109 competent cells. Atleast 10 randomly selected white colonies were sequenced to determine the percentage of methylation status in normal and disease samples. Representative figure of the bisulfite converted followed by cloning and sequencing data for both disease and adjacent normal tissue DNA has been prepared using QUMA tool [53].

BSP products were also used as templates for qMSP to determine methylation fold change in disease compared to adjacent normal tissue. BSP and qMSP primers were designed using MethPrimer tool [54]. qMSP was carried out using SYBR green (iTaq Universal SYBR Green Supermix, Bio-Rad) on 7900HT Fast Real-Time PCR system (Applied Biosystems). Methylation fold change in disease tissue was calculated in comparison to normal as using 2^−∆∆Ct^ method as stated previously [55], with minor modifications. Instead of calculating the percent methylation from the 2^−∆∆Ct^, we used fold change. Primers for BSP and qMSPs are provided in Additional file 6: Table S2.

### Gene expression study

Seventeen paired samples were used to study the expression pattern of selected genes. Out of these, 9 samples overlapped with the samples used for Illumina 450k methylation array. Tissue samples were snap-frozen in liquid nitrogen and finely ground to powder. Total RNA was isolated using AllPrep DNA/RNA Mini Kit (Qiagen, Germany). RNA concentration and quality was checked with Nanodrop Spectrophotometer (Nanodrop Technologies, Wilmington, DE). One microgramme of total RNA was used for cDNA synthesis using Transcriptor First Strand cDNA synthesis kit (Roche). cDNA was diluted, and 10 ng was used in each reaction of qPCR analysis. Gene expression qPCR primers were obtained from whole transcriptome qPCR primer database available through UCSC browser [56] and were amplified using iTaq Universal SYBR Green Supermix (Bio-Rad) in 7900HT Fast Real-Time PCR system (Applied Biosystems). Gene expression was normalised to expression of RNaseP (RPP30). GAPDH was also used as endogenous control. However, since both the genes gave similar results, data for only RPP30 has been used here. Gene expression qPCR primer sequences are provided in Additional file 6: Table S3.

### Cell culture

HEK293T cells were cultured in DMEM-F12 (GlutaMAX) medium (ThermoFisher Scientific) with 10% fetal bovine serum (Gibco) and 1% antibiotic (Penicillin-Streptomycin, Gibco) and incubated in 5% CO_2_ incubator at 37 °C. Cells were seeded on 24-well culture plates and grown until 80–90% confluency and changed to reduced serum media (Opti-MEM, Invitrogen), and then, transient transfection was done with the recombinant pCpGL luciferase constructs using Lipofectamine (Lipofectamine 2000, Thermo Scientific). Media were replaced with complete media after 6 h of transfection.

### Luciferase reporter assay

Promoters with at least 3 differentially methylated sites were selected for luciferase reporter assay. Core promoter element (including the TSS) was cloned into the upstream MCS of CpG-less (pCpGL) luciferase vector (InvivoGen). Primers used for promoter cloning are presented in Additional file 6: Table S4. pCpGl vector does not contain any CpG sites within its backbone and includes Lucia luciferase gene. This is a synthetic form of secreted luciferase that utilises colenterazine as substrate. Promoter constructs (pCpGL-promoter) were transformed into *E. coli* GT115 (Invivogen) *pir* mutant strain that lacks Dcm methylase. Positive colonies selected with Zeocin resistance were cultured and verified by Sanger sequencing. One part of the amplified plasmid was completely methylated with CpG Methylase enzyme (M.SssI, NEB). Complete methylation was checked by digestion with methylation-sensitive restriction enzymes HpaII, AciI and HinP1I (all purchased from NEB) (Additional file 2: Figure S4). Around 200 ng of methylated and unmethylated versions of each construct and an empty vector were transfected into HEK293T cells in triplicate. Minimal promoter-pGL4.24 (Promega), which utilises luciferin substrate, was used as transfection control. Twenty-four hours after transfection, luciferase activity was measured on Glomax 20/20 luminometer (Promega). Lucia luciferase value was normalised to that of firefly luciferase, and promoter activity of the constructs was calculated as fold change over the activity of M.SssI treated and untreated empty vector.

### Histological analysis

Formalin-fixed samples were cut into 2-mm thickness, and the slices were embedded in paraffin. Thin slices of 4-μm thickness were made from formalin-fixed paraffin embedded (FFPE) blocks using a microtome. After being mounted on poly-l-lysine-coated slides, and air dried, each slide was stained for haematoxylin and eosin (H&E) and covered with a cover slip using DPX mounting media and left to dry before image capture. The H&E-stained slides were used to assess overall abnormality of histopathology, including rete peg elongation, the presence of Munro’s microabscesses, Kogoj’s microabscesses and focal hypergranulosis (Additional file 2: Figure S5). A part of the normal tissues were also analysed histopathologically, and only histopathologically normal tissues were included in the study.

Among the 24 psoriasis samples studied in discovery set, all had characteristic histopathological features but varied only in terms of degree of rete peg elongation, the presence of Munro’s microabscesses, Kogoj’s microabscesses and focal hypergranulosis. In case of Munro’s microabscess, 11 samples were positive, 10 samples did not show the feature, while 3 samples could not be studied due to loss of stratum corneum during histopathological processing. The presence of granular layer along with focal hypergranulosis was observed in 7 samples, while others lacked this feature (Table 3). As in most of the cases, there are comparable numbers of patients with and without a specific histopathological feature; in the combined analysis, some of the feature-associated DMPs might have averaged out. So a separate paired differential methylation analysis was conducted between present and absent groups, for each histopathological feature. Unique sites were defined as those which were atleast 15% differential in either present or absent group, but not significantly different in the counterpart. Pearson’s correlation coefficient between the methylation status (*β* values) of 4133 DMPs and maximum rete peg length was carried out using cor.test function in R. Probes with correlation *P* value ≤ 0.05 were considered for further analysis.

### GO enrichment

To functionally characterise the genes that were differentially methylated, we tested for enrichment of GO terms with DAVID GO (https://david.ncifcrf.gov/). Gene ontology was done using genes that had overlapped with the differentially methylated probes (DMPs) at their promoters (from 1000 bp upstream to the + 500 bp downstream with respect to the TSS) [57, 58]. A term was identified as significant if the adjusted *P* value was ≤ 0.05. For the unique DMPs in Munro’s microabscess, we presented the unique GO terms with a cut-off enrichment score ≥ 1.5 and *P* value ≤ 0.05.

### Statistical analysis

Statistical tests were performed in R, unless otherwise mentioned. Spearman rank correlation coefficient was determined for the gene expression fold change and methylation (*β* values). The Fisher exact test was conducted for comparisons with small sample size. All *P* values were adjusted using Benjamini-Hochberg multiple hypothesis testing correction, and adjusted *P* value ≤ 0.05 was considered to be significant.

## Additional files

Additional file 1: List of total 4133 DMPs identified in the study. (XLSX 706 kb)

Additional file 2: **Figure S1.** Characterisation of DMPs. (a) Distribution of DMPs across different genomic regions; separate study of localization of hyper- and hypomethylated DMPs. (b) Figure showing comparison of frequency of DMPs and total probes in 450 k array across different genomic regions. *Indicates significantly enriched regions according to hypergeometric test. (c) Overlap of DMPs across regions containing repeat elements, (d) their distribution over genomic regions and (e) sub-classification into different types of repeat elements. **Figure S2.** Characterisation of differential CGI. (a) Hierarchical clustering of differential CGI. Normal samples are marked in light green and disease samples are marked with dark green. (b) Distribution of DMPs over CGI and associated regions (shores and shelves) and non-CGI regions (Open Sea); separate classification of hyper- and hypomethylated DMPs across CGI and associated regions. (c) Extent of hyper- and hypomethylation over CGI and associated shores and shelves. **Figure S3.** Characterisation of differential promoters. (a) Heatmap of differentially methylated promoters. Normal samples are marked in light green and disease samples are marked with dark green. (b) Classification of differential promoters into hyper- and hypomethylation; overlap of hyper- and hypomethylayed promoters with CGI and associated regions and non-CGI regions. (c) Validation of candidate promoter methylation in additional samples by qMSP. (d) Gene expression analysis of corresponding genes between disease and adjacent normal samples. **Figure S4.** Verification of complete methylation of promoter-pCpGL constructs. Unmethylated and methylated promoter constructs were digested with methylation sensitive restriction enzymes (HinP1I, AciI, HpaII) and run on 1% agarose gel. Lane1: 100 bp ladder; 2: SELENBP1 unmethylated uncut construct; 3: SELENBP1 unmethylated cut; 4: SELENBP1 methylated cut; Lane5: 100 bp ladder; 6: S100A9 unmethylated uncut construct; 7: S100A9 unmethylated cut; 8: S100A9 methylated cut. **Figure S5.** Description of Histopathological features studied :(a) × 10 magnification images of histopathological sections of adjacent normal and psoriatic disease tissue samples, (b) × 40 magnification image of stratum corneum in samples with and without Munro’s microabscess. (c) × 40 magnification image of stratum corneum and stratum spinosum junction showing Kogoj’s microabscess and focal hypergranulosis. **Figure S6.** Correlation of methylation status of sites with *R* > 0.6 with rete peg length. (a) Heatmap of sites correlated (*r* > 0.6) with rete peg elongation. (b) PCA with highly correlated (*R* > 0.7) sites. (PDF 1185 kb)

Additional file 3: List of differentially methylated promoters that overlap with PSORS loci. Significantly (hypergeometric test) enriched PSORS loci are presented in bold. (XLSX 29 kb)

Additional file 4: Total list of differentially methylated promoters. Hypo- and hypermethylated promoters are presented in two sheets. (XLSX 86 kb)

Additional file 5: List of top enriched biological processes (including the list of genes involved in each process) identified from gene ontology analysis of differentially methylated promoters (Table S1) and unique differentially methylated promoters in Munro’s microabscess (Table S2). (XLSX 13 kb)

Additional file 6 Table S1. List of biological processes enriched in gene ontology study for the hypermethylated and hypomethylated promoters. Table S2. List of primers used for BSP and qMSP validation. Table S3. List of primers used for gene expression study. Table S4. List of primers used for cloning promoter regions. (DOCX 21 kb)

Additional file 7: List of unique and common differential probes obtained on classification of samples with and without Munro’s microabscess. (XLSX 544 kb)

Additional file 8: List of probes correlated with rete peg length. (XLSX 44 kb)

Additional file 9 List of unique and common differential probes obtained on classification of samples with and without focal hypergranulosis. (XLSX 972 kb)

Additional file 10: List of unique and common differential probes obtained on classification of samples with and without Kogoj’s microabscess. (XLSX 672 kb)

## Abbreviations

ADAM10: A disintegrin and metalloproteinase domain-containing protein 10
AIF1: Allograft inflammatory factor 1
BMIQ: Beta-mixture quantile
BSP: Bisulfite sequencing PCR
CARD14: Caspase recruitment domain family member 14
CCL25: C-C motif chemokine ligand 25
CEBPA: CCAAT/enhancer binding protein alpha
CGI: CpG island
ChAMP: Chip Analysis of Methylation Pipeline
DENND1C: DENN domain containing 1C
DMPs: Differentially methylated probes
DNMT1: DNA methyltransferase 1
FDR: False discovery rate
FFAR2: Free fatty acid receptor 2
FFPE: Formalin-fixed paraffin embedded
GJC2: Gap junction protein gamma 2
GO: Gene ontology
GPR128: G protein-coupled receptor 128
H&E: Haematoxylin and eosin
HLA-DMA: Human leukocyte antigen-DMA
HRH1: Histamine receptor H1
ID4: Inhibitor of DNA binding 4
IL1B: Interleukin 1 beta
KAZN: Kazrin
LAMA4: Laminin subunit alpha 4
LINE-1: Long interspersed nuclear elements 1
MST1R: Macrophage stimulating 1 receptor
NDUFB4: NADH dehydrogenase 1 beta subcomplex 4
OAS2: 2′-5′-Oligoadenylate synthetase 2
PCA: Principal component analysis
pCpGL: CpG-less luciferase reporter vector
PDE4D: Phosphodiesterase 4D
PSORS: Psoriasis susceptibility
PTPN22: Protein tyrosine phosphatase non-receptor type 22
PTPN6: Protein tyrosine phosphatase non-receptor 6
qMSP: Quantitative methylation-specific PCR
RPP30: RNase P
S100A3, S100A5, S100A13, S100A8, S100A9, S100A12: S100 calcium-binding genes
SELENBP1: Selenium-binding protein
SFRP4: Secreted frizzled-related protein 4
SYNGR1: Synaptogyrin 1
TNXB: Tenascin-XB
TREM1: Triggering receptor expressed on myeloid cells 1
TSS: Transcription start site
TSSK1B: Testis-specific serine kinase 1B
ZNF106: Zinc finger protein 106
