# Supplementary material for: Epigenome-wide DNA methylation regulates cardinal pathological features of psoriasis
Source: Clin Epigenetics. 2018 Aug 9;10:108. doi: 10.1186/s13148-018-0541-9 (PMC6085681; doi:10.1186/s13148-018-0541-9)
Supplement: Supplementary file 2 — Figure S1. Characterisation of DMPs. (a) Distribution of DMPs across different genomic regions; separate study of localization of hyper- and hypomethylated DMPs. (b) Figure showing comparison of frequency of DMPs and total probes in 450 k array across different genomic regions. *Indicates significantly enriched regions according to hypergeometric test. (c) Overlap of DMPs across regions containing repeat elements, (d) their distribution over genomic regions and (e) sub-classification into different types of repeat elements. Figure S2. Characterisation of differential CGI. (a) Hierarchical clustering of differential CGI. Normal samples are marked in light green and disease samples are marked with dark green. (b) Distribution of DMPs over CGI and associated regions (shores and shelves) and non-CGI regions (Open Sea); separate classification of hyper- and hypomethylated DMPs across CGI and associated regions. (c) Extent of hyper- and hypomethylation over CGI and associated shores and shelves. Figure S3. Characterisation of differential promoters. (a) Heatmap of differentially methylated promoters. Normal samples are marked in light green and disease samples are marked with dark green. (b) Classification of differential promoters into hyper- and hypomethylation; overlap of hyper- and hypomethylayed promoters with CGI and associated regions and non-CGI regions. (c) Validation of candidate promoter methylation in additional samples by qMSP. (d) Gene expression analysis of corresponding genes between disease and adjacent normal samples. Figure S4. Verification of complete methylation of promoter-pCpGL constructs. Unmethylated and methylated promoter constructs were digested with methylation sensitive restriction enzymes (HinP1I, AciI, HpaII) and run on 1% agarose gel. Lane1: 100 bp ladder; 2: SELENBP1 unmethylated uncut construct; 3: SELENBP1 unmethylated cut; 4: SELENBP1 methylated cut; Lane5: 100 bp ladder; 6: S100A9 unmethylated uncut construct; 7: S100A9 unmethylated c [file 13148_2018_541_MOESM2_ESM.pdf]

Additional File 1

Figure S1.

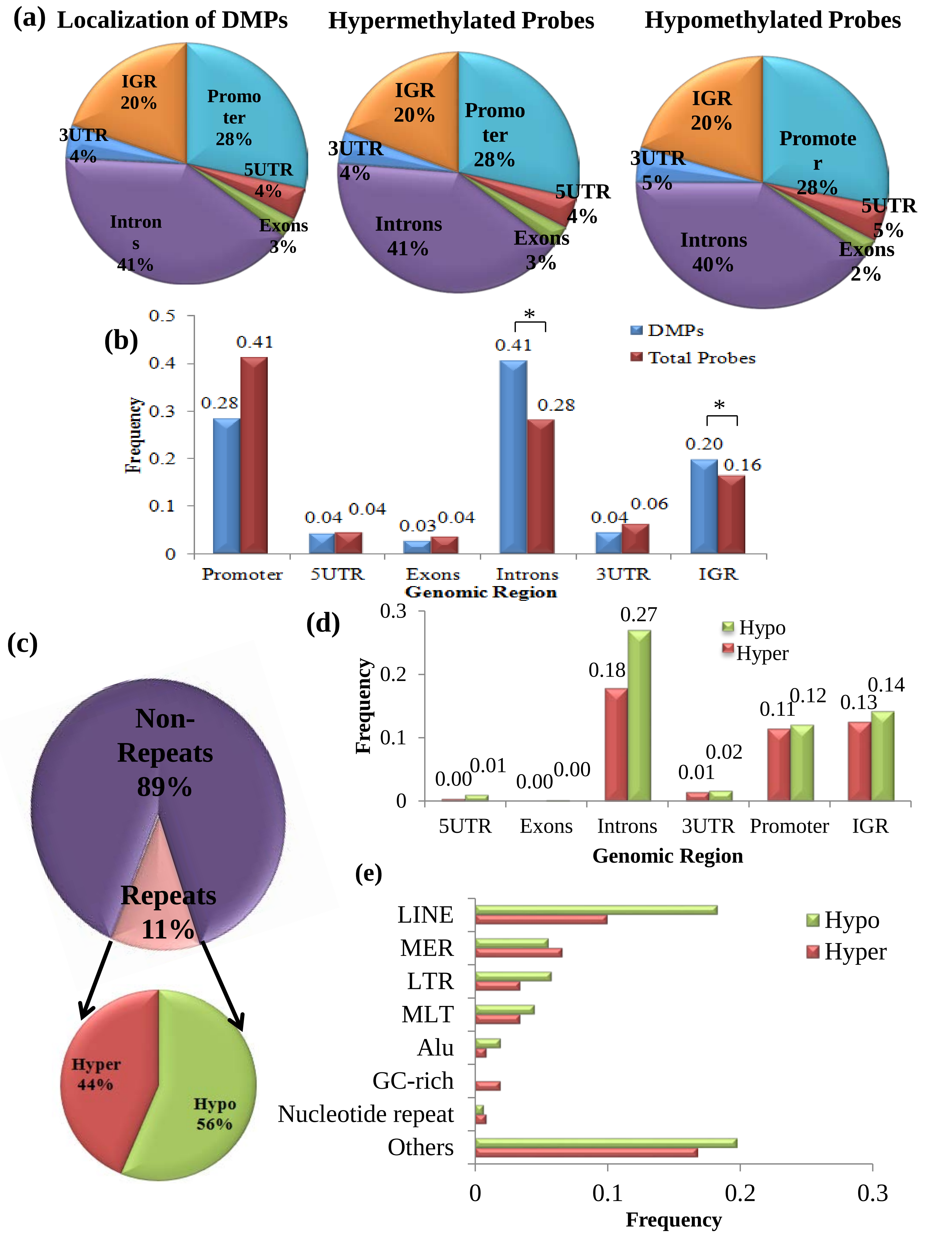

Figure S2.

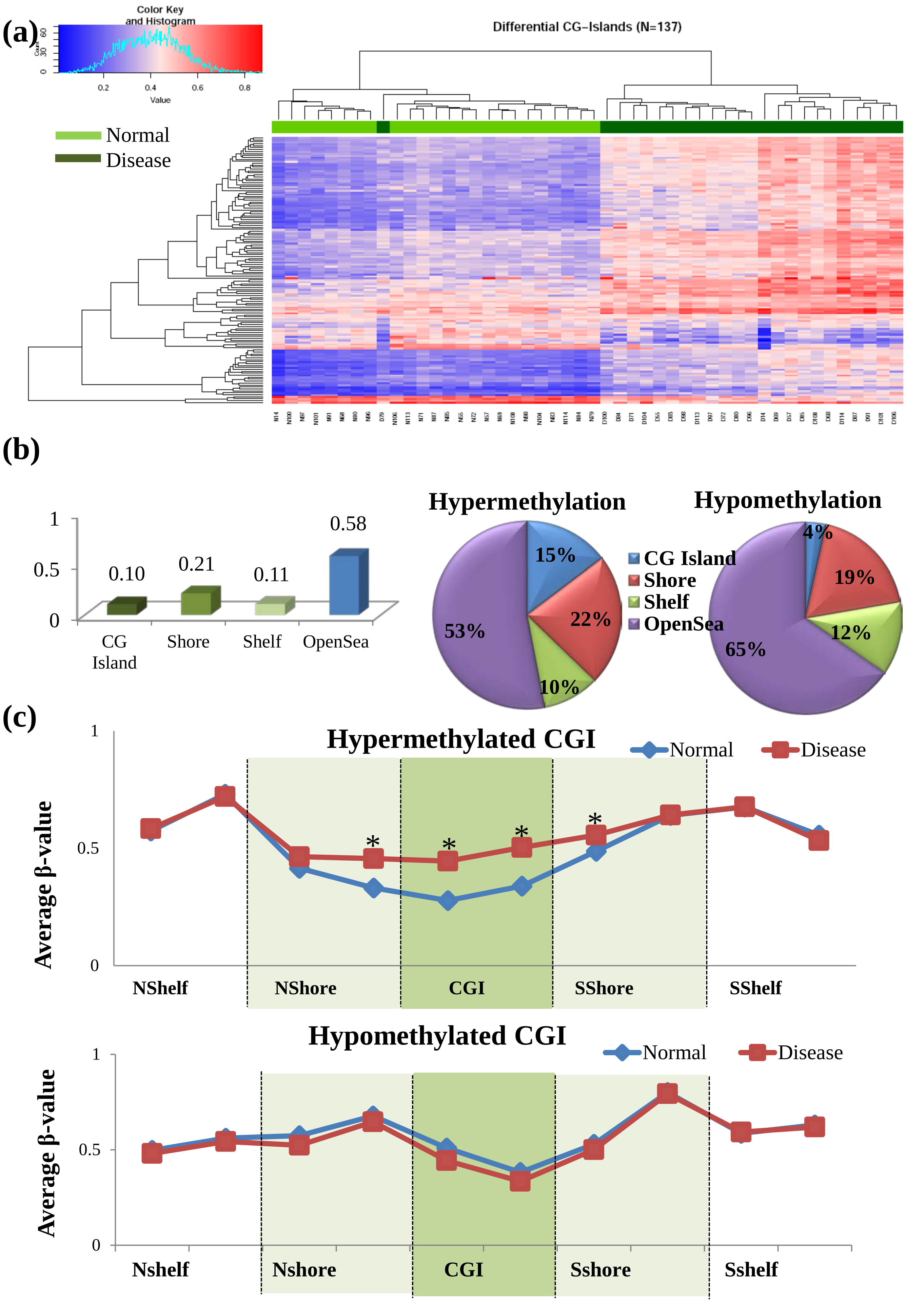
(a)
Color Key
and Histogram
Count
Value
0.2
0.4
0.6
0.8
Differential CG-Islands (N=137)
Normal
Disease
(b)
1
0.5
0
0.10
0.21
0.11
0.58
CG Island
Shore
Shelf
OpenSea
Hypermethylation
15%
22%
10%
53%
Hypomethylation
4%
19%
12%
65%
CG Island
Shore
Shelf
OpenSea
(c)
Hypermethylated CGI
Normal
Disease
Average β-value
1
0.5
0
*
NShelf
NShore
CGI
SShore
SShelf
Hypomethylated CGI
Normal
Disease
Average β-value
1
0.5
0
Nshelf
Nshore
CGI
Sshore
Sshelf

Figure S3.

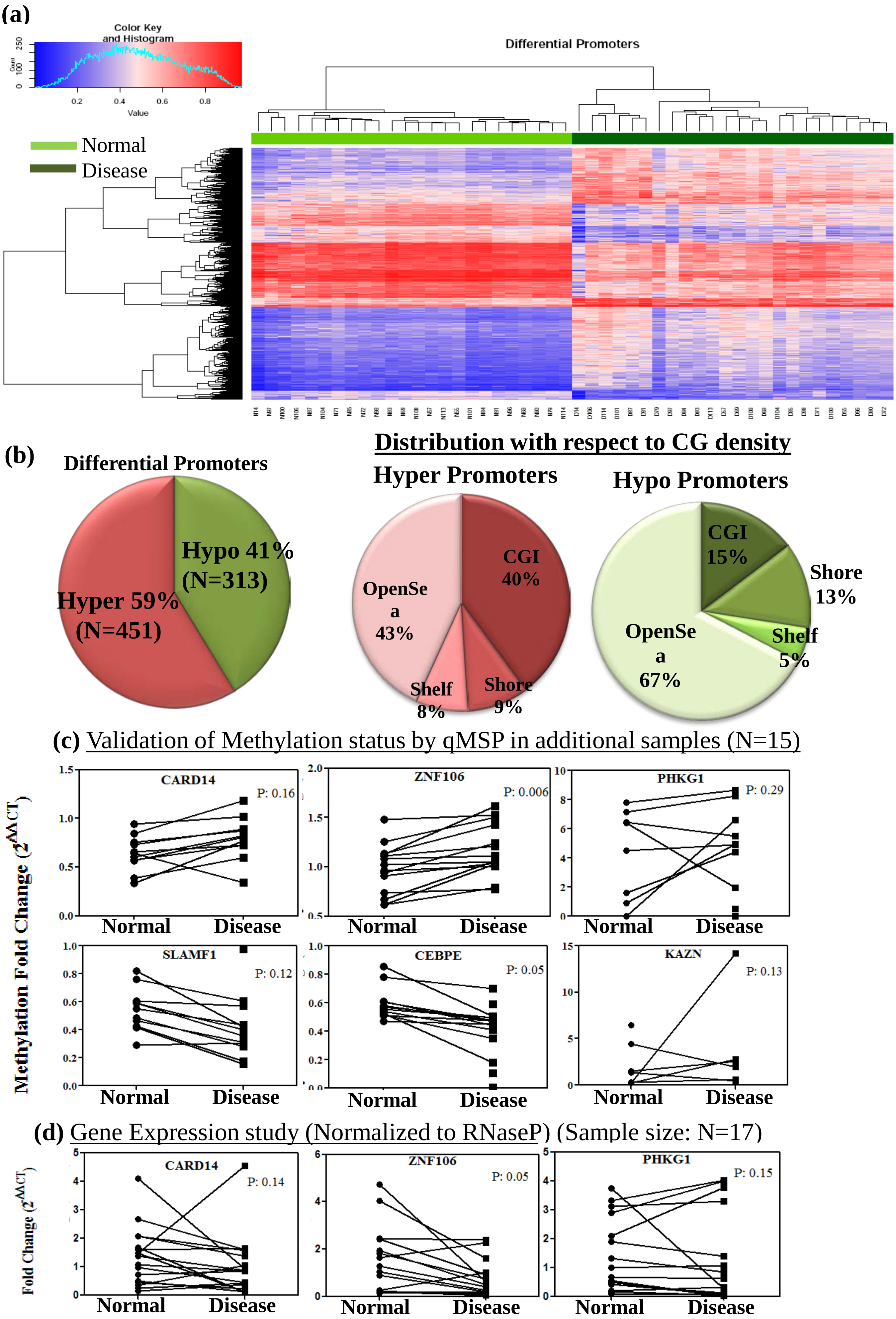

(a)
Color Key and Histogram
Count
Value
Differential Promoters
Normal
Disease
(b)
Differential Promoters
Hypo 41% (N=313)
Hyper 59% (N=451)
Distribution with respect to CG density
Hyper Promoters
CGI 40%
OpenSea 43%
Shelf 8%
Shore 9%
Hypo Promoters
CGI 15%
Shore 13%
Shelf 5%
OpenSea 67%
(c) Validation of Methylation status by qMSP in additional samples (N=15)
Methylation Fold Change ($2^{-\Delta\Delta CT}$)
CARD14 P: 0.16
ZNF106 P: 0.006
PHKG1 P: 0.29
SLAMF1 P: 0.12
CEBPE P: 0.05
KAZN P: 0.13
Normal
Disease
(d) Gene Expression study (Normalized to RNaseP) (Sample size: N=17)
Fold Change ($2^{-\Delta\Delta CT}$)
CARD14 P: 0.14
ZNF106 P: 0.05
PHKG1 P: 0.15
Normal
Disease

Figure S4.

Verification of complete methylation of promoter-pCpGL constructs

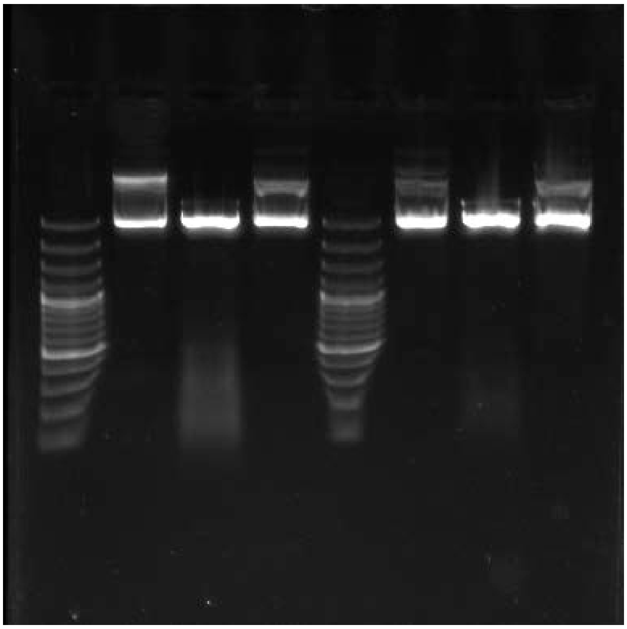

Lane1: 100bp ladder; 2: SELENBP1 unmethylated uncut construct; 3: SELENBP1 unmethylated cut; 4: SELENBP1 methylated cut

Lane5: 100bp ladder; 6: S100A9 unmethylated uncut construct; 7: S100A9 unmethylated cut; 8: S100A9 methylated cut

Figure S5.

(a)

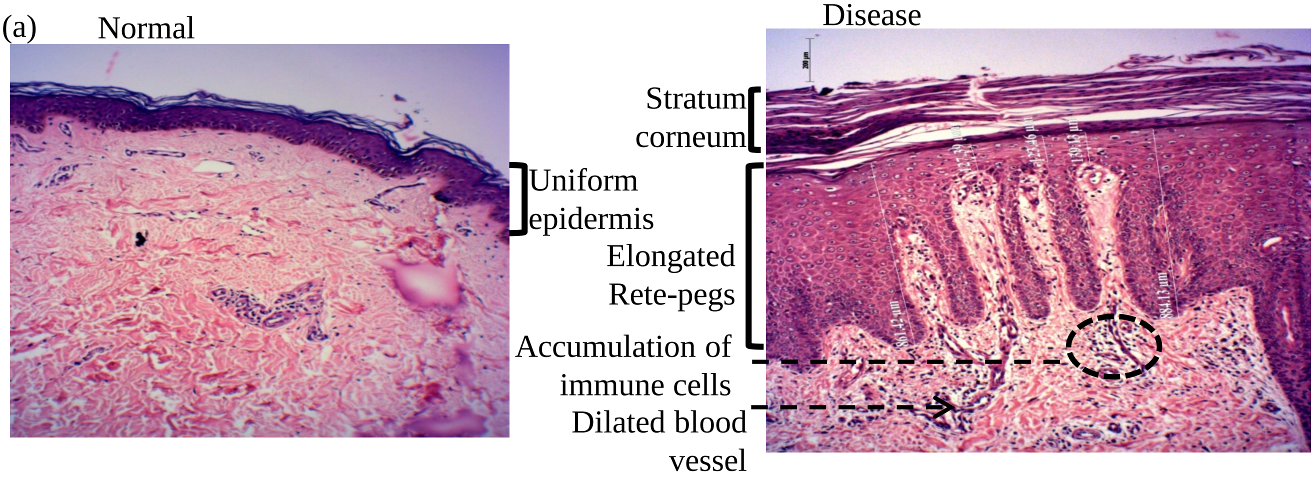

(b) 40X magnification image of Stratum Corneum

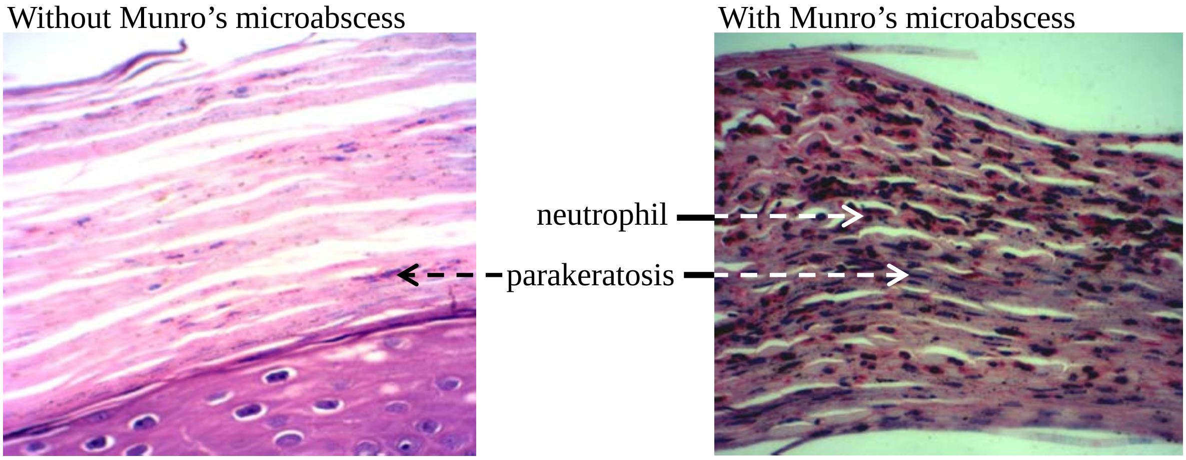

(c) 40X magnification image of junction of Stratum Corneum and Stratum Spinosum

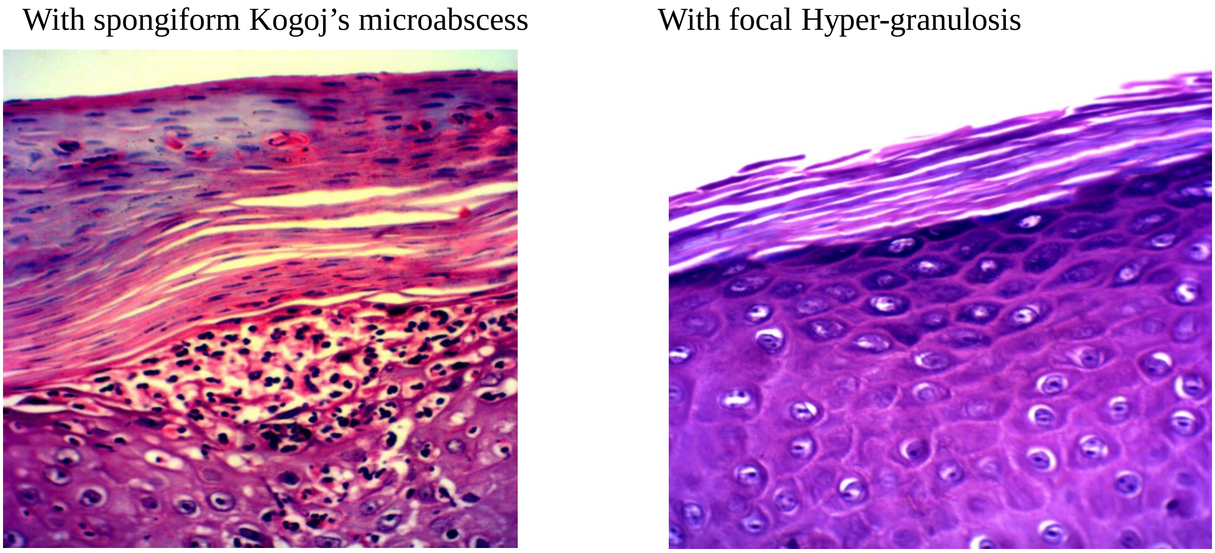

Figure S6.

(a)

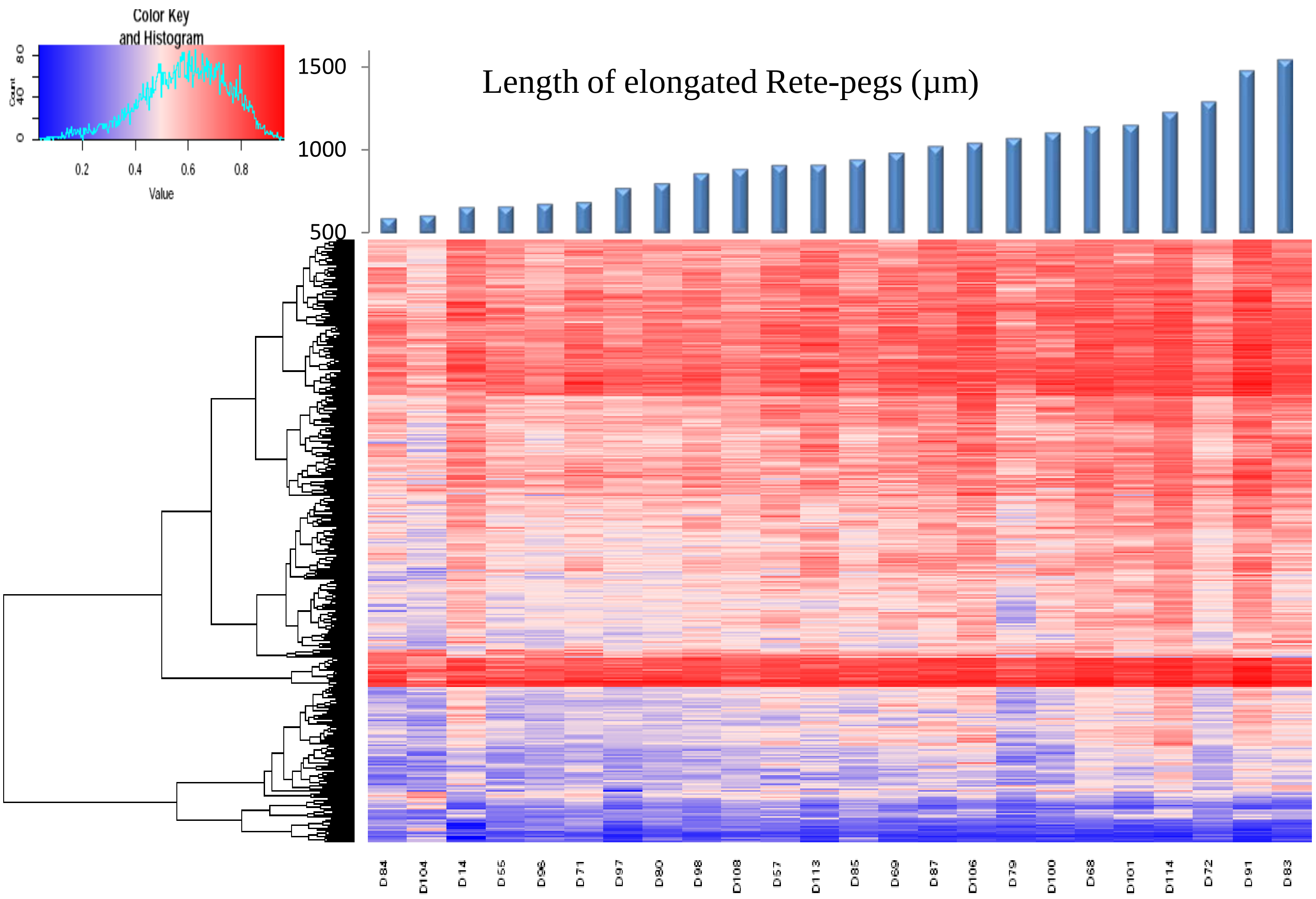

Color Key and Histogram
Count
Value
Length of elongated Rete-pegs (μm)
1500
1000
500
D84
D104
D14
D55
D96
D71
D97
D80
D98
D108
D57
D113
D85
D69
D87
D106
D79
D100
D68
D101
D114
D72
D91
D83

(b)

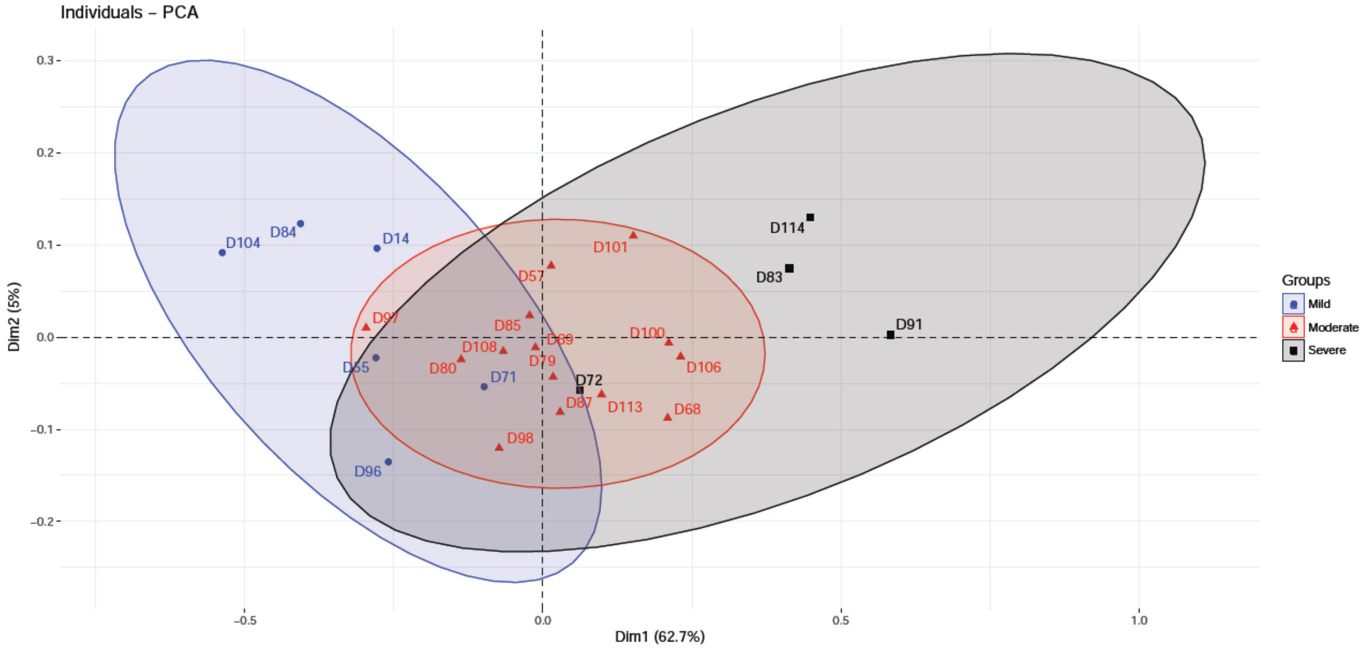

Individuals – PCA
Dim2 (5%)
Dim1 (62.7%)
Groups
Mild
Moderate
Severe
