# Supplementary material for: Epigenome-wide DNA methylation regulates cardinal pathological features of psoriasis
Source: Clin Epigenetics. 2018 Aug 9;10:108. doi: 10.1186/s13148-018-0541-9 (PMC6085681; doi:10.1186/s13148-018-0541-9)
Supplement: Supplementary file 6 — Table S1. List of biological processes enriched in gene ontology study for the hypermethylated and hypomethylated promoters. Table S2. List of primers used for BSP and qMSP validation. Table S3. List of primers used for gene expression study. Table S4. List of primers used for cloning promoter regions. (DOCX 21 kb) [file 13148_2018_541_MOESM6_ESM.docx]

**Additional File 4**

**Table S1**

| **Gene Ontology for the Hypermethylated Promoters** | | |  |
| --- | --- | --- | --- |
| **GO_Term: Biological Process** | **Gene Count (%)** | **P-value** | **Fold Enrichment** |
| cell motility | 11.88 | 3.44×10^-5^ | 1.87 |
| cell migration | 10.64 | 8.76×10^-5^ | 1.88 |
| cell adhesion | 12.13 | 4.27×10^-3^ | 2.26 |
| extracellular matrix organization | 4.46 | 2.73×10^-4^ | 2.79 |
| response to endogenous stimulus | 11.88 | 1.11×10^-3^ | 1.61 |
| actin cytoskeleton organization | 6.93 | 9.57×10^-6^ | 2.62 |
| developmental process | 33.42 | 1.58×10^-3^ | 1.24 |
| regulation of developmental process | 14.60 | 3.70×10^-3^ | 1.44 |
| cell differentiation | 21.04 | 3.76×10^-2^ | 1.21 |
| regulation of GTPase activity | 6.19 | 3.92×10^-3^ | 1.88 |
|  |  |  |  |
| **Gene Ontology for the Hypomethylated Promoters** | | |  |
| **GO_Term: Biological Process** | **Gene Count (%)** | **P-value** | **Fold Enrichment** |
| regulation of immune system process | 25.56 | 4.55×10^-22^ | 3.70 |
| intracellular signal transduction | 25.19 | 1.08×10^-7^ | 1.89 |
| immune response-regulating cell surface receptor signaling pathway | 8.15 | 1.10×10^-7^ | 4.08 |
| cytokine production | 12.22 | 9.99×10^-11^ | 3.87 |
| inflammatory response | 13.33 | 1.22×10^-12^ | 4.18 |
| response to stress | 35.19 | 1.81×10^-11^ | 1.91 |
| lymphocyte aggregation | 12.22 | 9.60×10^-15^ | 5.46 |
| cell proliferation | 14.81 | 3.33×10^-3^ | 1.59 |
| T cell activation via T cell receptor contact with antigen bound to MHC molecule on antigen presenting cell | 1.85 | 2.14×10^-6^ | 46.23 |
| inflammatory response | 13.33 | 1.22×10^-12^ | 4.18 |

**Table S2:** **BSP and qMSP Primers**

| **Primer Name** | **Purpose** | **Sequence** |
| --- | --- | --- |
| CARD14_BSP_F | BSP | GATTATTGTAGGAGTTGGGAATTTG |
| CARD14_BSP_R | BSP | AAACAACAACCCAAAAAAAACTTAA |
| CARD14_MSP_M_F | qMSP | GGTTATAGTTTAGGGGTGAGTATCG |
| CARD14_MSP_M_R | qMSP | AACCCAAAAAAAACTTAAAACTCG |
| CARD14_MSP_U_F | qMSP | GTTATAGTTTAGGGGTGAGTATTGG |
| CARD14_MSP_U_R | qMSP | ACCCAAAAAAAACTTAAAACTCAAA |
| CEBPE_BSP_F | BSP | GGGTTTGGTTAAAGTATTTGGTTAG |
| CEBPE_BSP_R | BSP | AAAAAAAATAAACCCAAAAAACAC |
| CEBPE_MSP_M_F | qMSP | CGGAGTAGATAGTTATAATTTTTCGG |
| CEBPE_MSP_M_R | qMSP | AAAAAAATAAACCCAAAAAACACG |
| CEBPE_MSP_U_F | qMSP | TGTTTTTGGAGTAGATAGTTATAATTTTTT |
| CEBPE_MSP_U_R | qMSP | AAAAAAATAAACCCAAAAAACACAC |
| DENND1C_BSP_F | BSP | TAGGTTTTTTTGAGGATTAGAGAGT |
| DENND1C_BSP_R | BSP | CTCCTAACTAATTAATCCCCCTACC |
| DENND1C_MSP_M_F | qMSP | GGGTTTTTGGATTTTGTATTTTC |
| DENND1C_MSP_M_R | qMSP | TTCCTACTTCGTAACCCCGA |
| DENND1C_MSP_U_F | qMSP | GGGTTTTTGGATTTTGTATTTTTG |
| DENND1C_MSP_U_R | qMSP | AACTTTCCTACTTCATAACCCCAA |
| S100A9_BSP_F | BSP | GTTTGTTTTAATTTTAAAGGGGATG |
| S100A9_BSP_R | BSP | ACACTTAACCCTTTAACCCTATCTC |
| S100A9_MSP_M_F | qMSP | GTTTTGTGAATATTTTTTGTTTCGT |
| S100A9_MSP_M_R | qMSP | TTAACCCTTTAACCCTATCTCCG |
| S100A9_MSP_U_F | qMSP | GTTTTGTGAATATTTTTTGTTTTGT |
| S100A9_MSP_U_R | qMSP | ACTTAACCCTTTAACCCTATCTCCA |
| SELENBP1_BSP_F | BSP | GTAATGGGTGATTGGAAATTTTTT |
| SELENBP1_BSP_R | BSP | CCCACCCCCAACTAATTATATAAAT |
| SELENBP1_MSP_M_F | qMSP | TTGTCGATTGGTATATTTTGATTTC |
| SELENBP1_MSP_M_R | qMSP | ACTAATTATATAAATTCCCTCCCTTCG |
| SELENBP1_MSP_U_F | qMSP | TTGTTGATTGGTATATTTTGATTTTG |
| SELENBP1_MSP_U_R | qMSP | ACTAATTATATAAATTCCCTCCCTTCACT |
| ZNF106_BSP_F | BSP | AGGGTAGGGGGTTTAGAGTAAGTAG |
| ZNF106_BSP_R | BSP | ATAAATCACATATATCATCCAAACCAA |
| ZNF106_MSPMF | qMSP | TGTATTTAGAAGGTTTGGTGATAGC |
| ZNF106_MSPMR | qMSP | GATTTAATTACCGACTTAAAATTAACTACG |
| ZNF106_MSPUF | qMSP | TATTTAGAAGGTTTGGTGATAGTGT |
| ZNF106_MSPUR | qMSP | AATTTAATTACCAACTTAAAATTAACTACA |
| SLAMF1_BSP_F | BSP | GGTTGTTTGATTTATGTATTAGGAATTAGT |
| SLAMF1_BSP_R | BSP | AAAATAATCCAATAAAACATCTTTTCTTTA |
| SLAMF1_MSP_M_F | qMSP | GGTTTTGGGTAGAAATATGCGT |
| SLAMF1_MSP_M_R | qMSP | TACAATCCAAAAAAACTTACCGAA |
| SLAMF1_MSP_U_F | qMSP | GGTTTTGGGTAGAAATATGTGT |
| SLAMF1_MSP_U_R | qMSP | CTTTACAATCCAAAAAAACTTACCAA |
| PTPN22_BSP_F | BSP | TTTTTTGGTTTATGTTGTAGAGTAAGAAA |
| PTPN22_BSP_R | BSP | AAAATAATCTCAATTAAACAAACCACACT |
| PTPN22_MSP_M_F | qMSP | TATTTTAAGAGTAATTTGGTTTCGG |
| PTPN22_MSP_M_R | qMSP | CTTCAACATACTCTACTCAAACGAC |
| PTPN22_MSP_U_F | qMSP | TATTTTAAGAGTAATTTGGTTTTGG |
| PTPN22_MSP_U_R | qMSP | CTTCAACATACTCTACTCAAACAAC |
| KAZN_BSP_F | BSP | TTGGTTTTTAGAGGGAGGATTTAGT |
| KAZN_BSP_R | BSP | AACAATATTTAAAACTCTCCCCACC |
| KAZN_MSP_M_F | qMSP | GTTTTTTTGAAAAATTTTAGATGGC |
| KAZN_MSP_M_R | qMSP | AATATTTAAAACTCTCCCCACCG |
| KAZN_MSP_U_F | qMSP | TTTTTTTGAAAAATTTTAGATGGTGT |
| KAZN_MSP_U_R | qMSP | ATATTTAAAACTCTCCCCACCAAA |
| PHKG1_BSP_F | BSP | TTTTTGGTTTTTAGGTTTAGAGAA |
| PHKG1_BSP_R | BSP | CTAAATATAAACCACTACACCC |
| PHKG1_MSP_M_F | qMSP | TTTTTGGTTTTTAGGTTTAGAGAAC |
| PHKG1_MSP_M_R | qMSP | TAATCATACACACTATCCCTACGAC |
| PHKG1_MSP_U_F | qMSP | TTTTTTGGTTTTTAGGTTTAGAGAATG |
| PHKG1_MSP_U_R | qMSP | TATAAAATAATCATACACACTATCCCTACA |

**Table S3: Gene expression primers**

| **Gene Name** | **Sequence** |
| --- | --- |
| e_CARD14_F | GAGCCGACAGGAGCTGGT |
| e_CARD14_R | GAACTGCAGCAGGGTCTGTT |
| e_CEBPE_F | CAGACAGCCATGCACCTG |
| e_CEBPE_R | GCCTTCTTGCCCTTGTGTAA |
| e_DENND1C_F | GGGTGCAGAGCCTTCTAATG |
| e_DENND1C_R | CCAAAGTGCTGAGTGATTGG |
| e_S100A9_F | AGGGGGAATTCAAAGAGCTG |
| e_S100A9_R | ATTTGTGTCCAGGTCCTCCA |
| e_SELENBP1_F | AGTGATGATCAGCTCCCTGG |
| e_SELENBP1_R | CAGGTCTCTCCCATGTCCC |
| e_SLAMF1_F | AGGAAGGAGGATGAGGGATG |
| e_SLAMF1_R | TTTCTGGAGTGGAGACCTGC |
| e_ZNF106_F | AGTATTCAGGCCGTGAGGCT |
| e_ZNF106_R | TTAGTGTGGTCGGTCAGCAA |
| e_PTPN22_F | GCTGTGGAAGGACTGGTGTT |
| e_PTPN22_R | TCCCGGATCAAACTGAAAAC |
| e_RPP30_F | CAATTTCCAGTGCCCTCAAT |
| e_RPP30_R | GCCTAGATTTGCCACGTCAT |
| e_PHKG1_F | AGCTCACAGACTTTGGCTTTTC |
| e_PHKG1_R | GTGGTCCTCATTCATGGAGC |

**Table S4: Promoter cloning primers**

| **Promoter_Name** | **Sequence** |
| --- | --- |
| CARD14_RE_F | TTTGAATCTGTGGACGGTGA |
| CARD14_RE_R | CAGCAAAGCTCCTGTGTTGA |
| S100A9_RE_F | GGGAGAGTAGGGCCTTAGGA |
| S100A9_RE_R | GGGAAGGAAAGAAGGAATCG |
| PTPN22_RE_F | GAGGGCATGTCAAATGTGTG |
| PTPN22_RE_R | ACCAGGTTCCTTTGCTGAGA |
| KAZN_RE_F | AGCCTCAAACACTGCTCGTT |
| KAZN_RE_R | CAGATAAAAAGCCCCAGCAA |
| ZNF106_RE_F | TCAGAGCAAGCAGAAGTTGC |
| ZNF106_RE_R | AAACACATTTGCTTGGCACA |
| SELENBP1_RE_F | CCAAATTAGGGGCAGACAGA |
| SELENBP1_RE_R | CATACACGCACACCCCATAA |
